# Disease progression and mortality with untreated HIV infection: evidence synthesis of HIV seroconverter cohorts, antiretroviral treatment clinical cohorts and population‐based survey data

**DOI:** 10.1002/jia2.25784

**Published:** 2021-09-21

**Authors:** Robert Glaubius, Nikhil Kothegal, Sehin Birhanu, Sasi Jonnalagadda, Severin Guy Mahiane, Leigh F. Johnson, Tim Brown, John Stover, Tara D. Mangal, Nikos Pantazis, Jeffrey W. Eaton

**Affiliations:** ^1^ Center for Modeling, Planning and Policy Analysis, Avenir Health Glastonbury Connecticut USA; ^2^ Public Health Institute/CDC Global Health Fellow Centers for Disease Control and Prevention Atlanta Georgia USA; ^3^ Division of Global HIV/AIDS Centers for Disease Control and Prevention Atlanta Georgia USA; ^4^ Centre for Infectious Disease Epidemiology and Research University of Cape Town Cape Town South Africa; ^5^ Research Program, East‐West Center Honolulu Hawaii USA; ^6^ MRC Centre for Global Infectious Disease Analysis School of Public Health Imperial College London London UK; ^7^ National and Kapodistrian University of Athens Medical School Athens Greece

**Keywords:** adults, cell counts, CD4, disease progression, HIV, statistical model, survival

## Abstract

**Introduction:**

Model‐based estimates of key HIV indicators depend on past epidemic trends that are derived based on assumptions about HIV disease progression and mortality in the absence of antiretroviral treatment (ART). Population‐based HIV Impact Assessment (PHIA) household surveys conducted between 2015 and 2018 found substantial numbers of respondents living with untreated HIV infection. CD4 cell counts measured in these individuals provide novel information to estimate HIV disease progression and mortality rates off ART.

**Methods:**

We used Bayesian multi‐parameter evidence synthesis to combine data on (1) cross‐sectional CD4 cell counts among untreated adults living with HIV from 10 PHIA surveys, (2) survival after HIV seroconversion in East African seroconverter cohorts, (3) post‐seroconversion CD4 counts and (4) mortality rates by CD4 count predominantly from European, North American and Australian seroconverter cohorts. We used incremental mixture importance sampling to estimate HIV natural history and ART uptake parameters used in the Spectrum software. We validated modelled trends in CD4 count at ART initiation against ART initiator cohorts in sub‐Saharan Africa.

**Results:**

Median untreated HIV survival decreased with increasing age at seroconversion, from 12.5 years [95% credible interval (CrI): 12.1–12.7] at ages 15–24 to 7.2 years (95% CrI: 7.1–7.7) at ages 45–54. Older age was associated with lower initial CD4 counts, faster CD4 count decline and higher HIV‐related mortality rates. Our estimates suggested a weaker association between ART uptake and HIV‐related mortality rates than previously assumed in Spectrum. Modelled CD4 counts in untreated people living with HIV matched recent household survey data well, though some intercountry variation in frequencies of CD4 counts above 500 cells/mm^3^ was not explained. Trends in CD4 counts at ART initiation were comparable to data from ART initiator cohorts. An alternate model that stratified progression and mortality rates by sex did not improve model fit appreciably.

**Conclusions:**

Synthesis of multiple data sources results in similar overall survival as previous Spectrum parameter assumptions but implies more rapid progression and longer survival in lower CD4 categories. New natural history parameter values improve consistency of model estimates with recent cross‐sectional CD4 data and trends in CD4 counts at ART initiation.

## 1 INTRODUCTION

HIV epidemic models, including the Joint United Nations Programme on HIV/AIDS (UNAIDS)‐supported Spectrum model [1], use data on HIV prevalence, HIV diagnoses or HIV‐related deaths to estimate HIV incidence trends [2, 3, 4, 5] and extrapolate estimates of HIV burden. Relationships between HIV incidence, prevalence and mortality are governed by natural history parameters of HIV infection, consisting of HIV disease progression and mortality rates among untreated individuals.

The assumptions of the Spectrum model are regularly updated to make the best possible use of new data. Previous disease progression and mortality rates implemented in Spectrum were estimated using data from observational cohort studies conducted before antiretroviral therapy (ART) was widely available [6]. Survival times after HIV seroconversion observed in East African seroconverter cohorts were a key data source for this estimation [7, 8]. These data inform average survival times but may not uniquely identify specific parameters, since similar survival times can result from faster disease progression and lower mortality rates or vice‐versa. Evidence from European and African seroconverter cohorts suggested that CD4 counts at seroconversion may be lower, and disease progression faster, than currently assumed in Spectrum [9, 10, 11].

Population‐based HIV Impact Assessment (PHIA) surveys [12, 13, 14, 15, 16, 17, 18, 19, 20, 21, 22] conducted in 10 sub‐Saharan African countries between 2015 and 2018 measured CD4 counts among nationally representative samples of untreated HIV‐positive adults. These data may improve our understanding of HIV natural history. Unlike data from the pre‐ART era, CD4 counts observed in untreated PHIA respondents are affected by ART uptake, which may informatively censor people who seek ART due to symptomatic HIV infection. Nevertheless, these data could help identify HIV disease progression and mortality rates among people living with HIV (PLHIV) who are not on ART and ensure that model‐based estimates reflect empirical CD4 distributions among untreated adults and at treatment initiation.

## 2 METHODS

We conducted a Bayesian evidence synthesis [23, 24, 25] to update HIV natural history inputs to Spectrum. We included cross‐sectional CD4 counts from ten PHIAs alongside previously analysed HIV progression and survival data. We distinguished “training data” included in our synthesis from data withheld for validation. We developed natural history equations to specify inputs for Spectrum, then evaluated the likelihood of training data by direct comparison to these inputs when possible. When direct comparison was not possible, we used models derived from Spectrum to calculate comparators. We describe these components below and in detail in Appendix S1.

### 2.1 Spectrum natural history and ART dynamics

**Figure 1:**
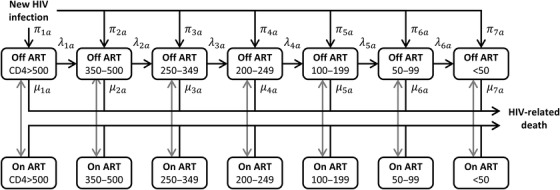
HIV infection stages in Spectrum. Spectrum model inputs specify the initial CD4 cell count distribution at infection ($\pi_{h,a}$), disease progression rates ($\lambda_{h,a}$) and HIV‐related mortality rates ($\mu_{h,a}$) by stage h and age group a (15–24, 25–34, 35–44 or 45+). Spectrum uses an ART allocation weight (ω, not shown) when calculating ART initiation; we did not estimate other inputs governing ART initiation and interruption (grey arrows) or HIV‐related mortality on ART in this analysis. Abbreviation: ART, antiretroviral therapy.

Spectrum and the EPP‐ASM sub‐model used to estimate HIV incidence [2, 26] share common demographic and epidemiologic structure and inputs [27]. Adult HIV disease progression is modelled as seven infection stages defined by CD4 cell count thresholds (CD4>500, 350 to 500, 250 to 349, 200 to 249, 100 to 199, 50 to 99 and <50 cells/mm^3^; Figure 1) that reflect historic ART initiation criteria and other clinically relevant values [27]. Newly infected adults enter stages (h) proportional to initial CD4 category distributions ($\pi_{h,a}$) stratified by age group a (15 to 24, 25 to 34, 35 to 44 or 45+). PLHIV progress sequentially through these stages ($\lambda_{h,a}$) and experience HIV‐related mortality ($\mu_{h,a}$) from each stage. PLHIV may exit untreated infection stages via background mortality or treatment initiation, and re‐enter upon treatment interruption. PLHIV who interrupt treatment return to their pre‐treatment infection stage (Spectrum was subsequently updated to model immune recovery on ART, per Stover et al. in this supplement) and resume pre‐treatment disease progression and mortality rates while off ART.

Treatment initiation rates are determined by country‐specific input data about numbers of adult ART patients by sex and year. Spectrum combines two approaches to allocate new ART patients among treatment‐eligible infection stages (per contemporary national guidelines): treatment initiation proportional to numbers eligible, or proportional to expected HIV‐related deaths in each stage, representing that those at higher mortality risk are more likely to have AIDS symptoms and seek care. Spectrum assigns weights ω to expected deaths and (1−ω) to numbers eligible to average these approaches. Country analysts can modify ω, but since this parameter is not observable, most assume the default ω=0.5 to give equal weight to both approaches.

### 2.2 Natural history model parameterization

We specified parametric models for the distribution of CD4 count following seroconversion, CD4 count decline rates and HIV‐related mortality by CD4 category, resulting in 11 parameters that determine the 80 natural history inputs in Figure 1. Parameters varied by four age groups a = 0, 1, 2 or 3, denoting 15–24, 25–34, 35–44 or 45+, respectively.

#### 2.2.1 Initial CD4 cell counts

We modelled initial CD4 counts using a log‐logistic distribution with age‐specific median $m_{a}$ and shape $\psi_{1}$. We specified the median directly for ages 15–24 ( $m_{0}=\psi_{2}\;$) then assumed medians decreased linearly with increasing age ($\psi_{3}$). The proportion of seroconverters with initial CD4 counts of c or less is as follows:

(1) $$P_{a}\;\left(C\leq c\right)=1/\left[1+{\left(\frac{c}{m_{a}}\right)}^{-\left(\psi_{1}+1\right)}\right]\;\;\mathrm{with}\;m_{a}=\psi_{2}\cdot \left(1-\psi_{3}a\right)$$

We derived Spectrum inputs $\pi_{h,a}$ directly from $P_{a}$.

#### 2.2.2 CD4 decline after seroconversion

We modelled CD4 counts as decreasing continuously over years t since seroconversion according to polynomial curves. We specified these curves $c_{a}\left(t\right)$ in terms of a baseline CD4 count $c_{a}\left(0\right)$, an age‐specific “depletion time” $\tau_{a}$ when CD4 counts reach zero cells/mm^3^ and shape parameter $\theta_{1}$. We specified depletion times relative to ages 15–24 ( $\tau_{0}=\theta_{2}\;$) that decreased linearly at older ages ($\theta_{3}$).

(2) $$c_{a}\;\left(t\right)=c_{a}\;\left(0\right){\left(1-\frac{t}{\tau_{a}}\right)}^{\theta_{1}+1}\;\mathrm{for}\;\tau_{a}=\theta_{2}\cdot \left(1-\theta_{3}a\right)$$

We defined baseline CD4 counts $c_{a}\left(0\right)$ as the median among seroconverters with initial CD4 counts over 500 cells/mm^3^ (Equation 1). We calculated progression rates $\lambda_{h,a}$ for Spectrum infection stages as the reciprocal of the time between crossing the upper and lower CD4 count thresholds of each stage.

#### 2.2.3 Mortality by CD4 cell count

We modelled HIV‐related mortality rates $\mu_{a}\left(c\right)$ that increase rapidly as CD4 counts c decline [5].

(3) $$\mu_{a}\left(c\right)=\varphi_{1}^{c}\cdot \varphi_{2}\cdot \left(1+\varphi_{3}a\right)\cdot \varphi_{4}$$

The shape parameter $\varphi_{1}$ controls how rapidly mortality increases as CD4 counts fall. Other parameters specify mortality rates among 15‐ to 24‐year‐olds with zero CD4 cells/mm^3^ ($\varphi_{2}$), a linear age effect ($\varphi_{3}$) and a mortality rate ratio ($\varphi_{4}$) that adjusts for potential bias in all‐cause mortality training data. We derived mortality rates $\mu_{h,a}$ for Spectrum infection stages as the average rate across CD4 counts in each stage.

#### 2.2.4 Treatment initiation

We assumed a non‐informative ω∼Uniform(0,1) prior on Spectrum's treatment allocation weight.

Prior distributions on parameters are provided in Table 2 and described in Appendix S1.

### 2.3 Training data

Our synthesis incorporated four data sources that each inform one or more natural history components. We formulated the joint likelihood as the product of likelihoods of each data source.

To inform initial CD4 counts, we included data on CD4 counts measured within 1 year of seroconversion compiled from 13,176 seroconverters in Europe, North America, Australia and sub‐Saharan Africa between 1982 and 2012 [9]. Data consisted of seroconverter counts tabulated by sex, CD4 category and 5‐year age group at seroconversion. We assumed that CD4 category frequencies were multinomially distributed conditional on age.

To inform HIV‐related mortality rates by CD4 category, we included all‐cause mortality data from the Concerted Action on SeroConversion to AIDS and Death in Europe (CASCADE) Collaboration [28] consisting of 12,679 person‐years of follow‐up and deaths observed in 1997–2004 among untreated HIV seroconverters by age and infection stage. Seroconversion date was identified from negative and first positive HIV tests done at most 3 years apart. We extracted data from the published report, which excluded people exposed to HIV via injection drug use. We assumed that observed deaths were Poisson‐distributed conditional on person‐years of follow‐up and all‐cause mortality rates, calculated as unadjusted (i.e. $\varphi_{4}=\;1$) HIV‐related mortality plus background mortality. We used age‐specific central mortality rate estimates for 1985–1990 Europe [29] weighted by sex (14% female) to approximate background mortality.

For overall HIV survival, we used individual‐level data from four population‐based cohort studies of HIV seroconverters from East Africa [7]. Individuals were followed during 1994–2004. Seroconversion dates were identified as the midpoint between last negative and first positive HIV tests done at most 4 years apart. Individuals exited at death or were censored at end of study or when last known alive. We included data from 1421 participants aged 15–59 at seroconversion. Overall survival depends on initial CD4 counts, disease progression, HIV‐related mortality and underlying non‐HIV mortality by age. Therefore, we calculated survival probabilities and mortality densities using a compartmental cohort model that implemented aging, background mortality and HIV natural history (Figure 1) as in Spectrum. Since most (70%) seroconverters were from Ugandan cohorts, we incorporated contemporary age‐ and sex‐specific Ugandan background mortality rates [8, 29].

We included data from 10 Africa‐based PHIAs conducted in 2015–2018 (Table 1). Data consisted of survey‐based estimates of nationally representative CD4 category distributions in untreated PLHIV by 5‐year age group (15–19 to 45–49). HIV‐positive survey respondents were classified as on ART if they reported ART use or had detectable antiretroviral drugs in blood, and off ART otherwise. These data inform initial CD4 counts, disease progression and HIV‐related mortality; they also inform ART uptake since some PLHIV may be “missing” because they started treatment. Since past HIV incidence, treatment scale‐up and demographic dynamics also affect these data, we used EPP‐ASM to calculate cross‐sectional CD4 count distributions from our natural history inputs. Remaining EPP‐ASM inputs were taken from national Spectrum files derived from the 2019 HIV estimates round [30]. We assumed that observed CD4 counts were multinomially distributed conditional on numbers of untreated HIV‐positive survey respondents and modelled age‐specific CD4 count distributions.

**Table 1:** Inclusion of HIV‐positive adults not on ART from Population‐based HIV Impact Assessment (PHIA) surveys

		15–49 years				
Country (survey years)	Eligible (age 15‐upper limit)^a^	Eligible	Participated in the PHIA interview	Participated in the PHIA blood draw and HIV testing	Tested HIV+ in the PHIA survey	HIV+ not on ART (based on self‐report and/or ARV detection)^b^
Cameroon (2017)	28,635	24,754	23,528	22,448	792	410
Côte d'Ivoire (2017–18)	21,312	18,628	16,653	15,688	337	231
Eswatini (2016–17)	12,857	10,164	9150	8536	2423	558
Malawi (2015–16)	22,405	20,036	17,490	15,258	1886	563
Namibia (2017)	21,464	18,446	16,056	14,463	1951	329
Nigeria (2018)	206,996	176,067	157,745	147,049	2208	1162
Tanzania (2016–17)	36,087	29,116	26,737	25,595	1455	627
Uganda (2016–17)	30,581	26,988	25,887	25,573	1488	504
Zambia (2015–16)	24,663	22,550	19,365	10,026	2131	792
Zimbabwe (2015–16)	25,131	21,508	19,109	17,466	2730	858

^a^ Eligible age‐range for adults to participate in the PHIA survey was 15–64 years in Cameroon, Côte d'Ivoire, Malawi, Namibia, Nigeria and Uganda; 15–59 in Zambia; and 15+ in Eswatini, Tanzania and Zimbabwe.

^b^ Excludes participants who tested HIV+ but were missing a CD4 cell count measurement.

Abbreviations: ARV, antiretroviral drug; PHIA, population‐based HIV impact assessment.

### 2.4 Validation data

We compared estimated CD4 counts at ART initiation, also calculated using EPP‐ASM, to data from ART initiators in IeDEA (International epidemiology Databases to Evaluate AIDS) collaborating clinical cohorts [31]. CD4 counts were measured within 3 months before ART initiation in previously ART‐naïve patients. We compared EPP‐ASM outputs to data from 471,220 ART initiators aged 16+ in seven countries (Côte d'Ivoire, Malawi, Nigeria, Tanzania, Uganda, Zambia and Zimbabwe), where PHIA data were also available. Since IeDEA sites may not be nationally representative, we aggregated ART initiators across countries. We weighted EPP‐ASM estimates by numbers of ART initiators at sites in each country. We restricted this comparison to data from 2005 to 2014, as data were not consistently available from these countries outside this period.

To determine whether natural history input estimates were appropriate outside sub‐Saharan Africa, we compared estimated survival times to published estimates in European, Australian and North American seroconverter cohorts [32]. These survival estimates included CASCADE cohort members and overlap with some of the training data about initial CD4 distribution and mortality by CD4 category.

### 2.5 Analyses

We used incremental mixture importance sampling (IMIS) [33] to sample posterior distributions on model parameters. We reported posterior mode point estimates and 95% central credible intervals about model parameters and outputs. We used the IMIS sample with maximal posterior density to approximate the posterior mode. We evaluated goodness‐of‐fit to training data graphically. We compared training and validation data to posterior predictive distributions on model outputs. In sensitivity analysis, we considered an alternate model that incorporated sex differences in disease progression and mortality via a rate ratio parameter subject to a Gamma(2,1) prior.

### 2.6 Ethics statement

Local ethics committees in each country and Institutional Review Boards at the US Centers for Disease Control and Prevention and Columbia University approved PHIA surveys. All participants provided written informed consent. Anonymized PHIA data were used for statistical analyses. Each programme participating in the IeDEA collaboration obtained ethical approval from relevant local institutions to collect and share patient data; in addition, each regional data centre obtained ethical approval to collate and analyse the de‐identified data. All collaborating cohorts in CASCADE received approval from their regulatory or national ethics review boards (Appendix S3). Separate ethical approval was not required for this study for data from ALPHA Network or Rwanda Maternal Child and Health studies, as these were secondary analyses of fully anonymized existing datasets [7].

## 3 RESULTS

### 3.1 Parameter estimates

**Figure 2:**
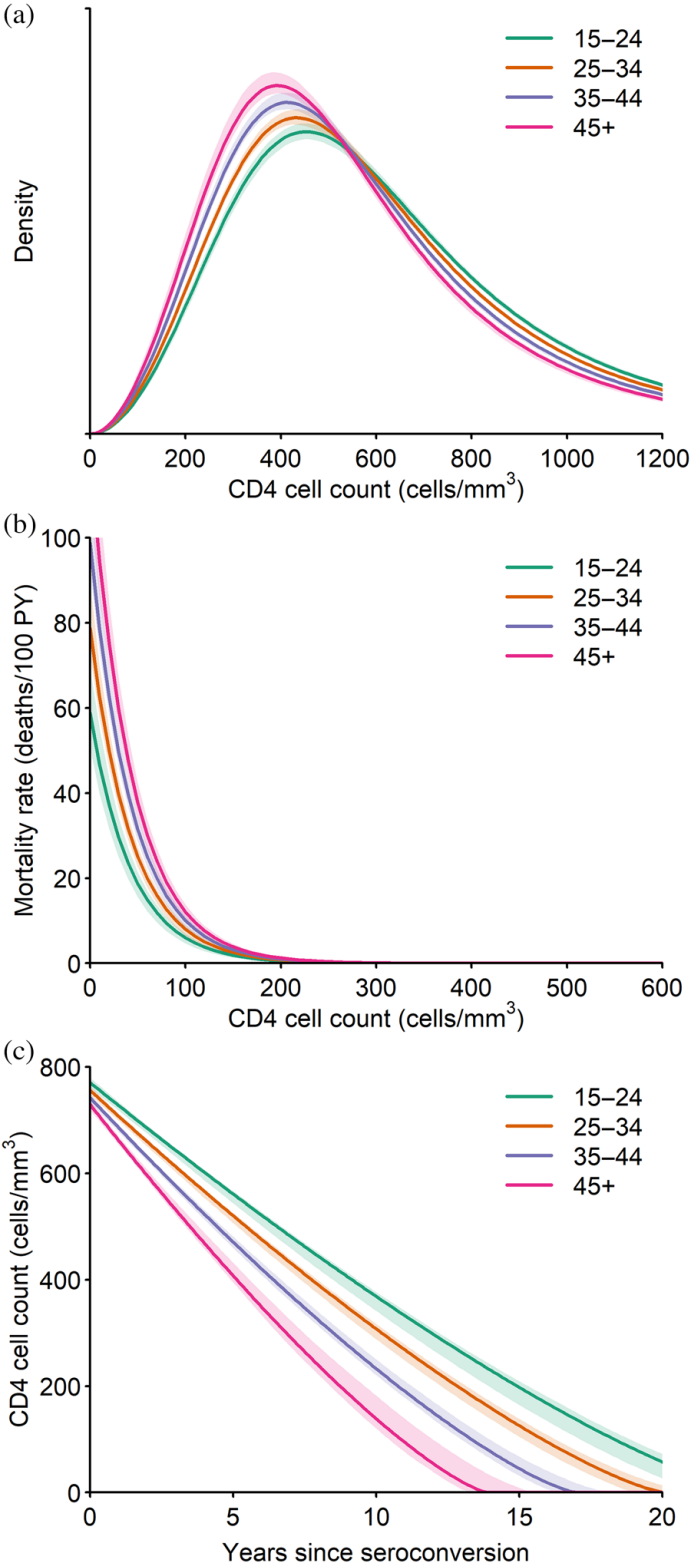
Fitted natural history parameter estimates by age. Panels show (a) initial CD4 cell count distributions, (b) HIV‐related mortality rates by CD4 cell count and (c) CD4 cell count trends. Posterior point estimates (solid curves) and 95% credible intervals (shaded regions) are shown.

The median CD4 count at seroconversion for persons aged 15–24 was estimated to be 579 cells/mm^3^ [95% credible interval (CrI): 570–589] and would decline to zero CD4 cells/mm^3^ 23 years (95% CrI: 21.5–24.4) after infection (Table 2). CD4 counts declined approximately linearly, but slopes decreased gradually as infection duration increased (Figure 2c). The ART allocation weight was 0.21 (95% CrI: 0.16–0.25), indicating more weight to numbers of people in each eligible infection stage and less weight to expected deaths in those stages, when calculating ART uptake. Table 3 reports corresponding Spectrum inputs; Appendix S2 compares our estimates to previous Spectrum assumptions.

**Table 2:** Natural history model parameters

Parameter	Symbol	Dimension	Prior	Posterior mode (95% CrI)
*Initial CD4 cell count*				
Shape parameter	$\psi_{1}$	…	Exp(1/3)	1.92 (1.86–1.99)
Age 15–24 median	$\psi_{2}$	CD4 cells/mm^3^	$\mathrm{Gamma}(\sqrt{585},\;\sqrt{585})$	579 (570–589)
Age effect	$\psi_{3}$	…	Uniform(0,1/3)	0.044 (0.036–0.055)
*CD4 cell count trend*				
Shape parameter	$\theta_{1}$	…	Exp(1)	0.31 (0.22–0.43)
Age 15–24 CD4 cell depletion time	$\theta_{2}$	years	Lognormal(3,0.29)	23.2 (21.5–24.4)
Age effect	$\theta_{3}$	…	Uniform(0,1/3)	0.133 (0.104–0.142)
*HIV‐related mortality*				
Shape parameter	$\varphi_{1}$	…	Beta(252,4)	0.977 (0.975–0.979)
Age 15–24 mortality rate at 0 CD4 cells/mm^3^	$\varphi_{2}$	person‐year^–1^	Gamma(3.8,0.25)	0.59 (0.51–0.69)
Age effect	$\varphi_{3}$	…	Exp(1)	0.34 (0.23–0.50)
Mortality rate ratio	$\varphi_{4}$	…	Gamma(2,1)	2.03 (1.83–2.60)
*Treatment allocation*				
ART allocation weight	ω	…	Uniform(0,1)	0.21 (0.16–0.25)

Abbreviations: ART, antiretroviral therapy; CrI, credible interval.

**Table 3:** Natural history input estimates by age (95% credible intervals)

CD4 cells/mm^3^	15–24	25–34	35–44	45+
**Initial CD4 cell count distribution, %**				
>500	60.5 (59.4–61.7)	57.4 (56.4–58.0)	53.9 (52.7–54.7)	50.3 (48.2–51.6)
350–500	20.8 (20.2–21.3)	21.9 (21.4–22.4)	22.9 (22.4–23.5)	23.8 (23.3–24.6)
250–349	10.8 (10.4–11.1)	11.8 (11.6–12.2)	13.0 (12.8–13.5)	14.3 (13.9–15.1)
200–249	3.6 (3.5–3.8)	4.1 (4.0–4.2)	4.6 (4.5–4.8)	5.2 (4.9–5.5)
100–199	3.7 (3.5–3.9)	4.2 (4.0–4.4)	4.8 (4.6–5.1)	5.5 (5.2–6.0)
50–99	0.5 (0.5–0.6)	0.6 (0.5–0.6)	0.7 (0.6–0.7)	0.8 (0.7–0.9)
<50	0.1 (0.1–0.1)	0.1 (0.1–0.1)	0.1 (0.1–0.1)	0.1 (0.1–0.1)
**Progression rate to the next lower CD4 category when not on ART, events per 100 person‐years**				
>500	15.3 (14.9–16.6)	18.3 (17.9–19.4)	22.5 (21.7–23.6)	28.6 (26.4–30.0)
350–500	25.1 (24.4–27.0)	28.6 (27.7–29.9)	33.3 (31.7–34.3)	40.2 (36.4–41.9)
250–349	34.8 (33.5–37.3)	39.5 (38.1–41.3)	46.1 (43.7–47.6)	55.6 (49.9–57.9)
200–249	65.1 (62.0–70.1)	74.0 (70.3–77.6)	86.2 (80.7–89.4)	104.0 (93.0–108.0)
100–199	29.4 (27.5–32.2)	33.5 (31.0–35.7)	39.0 (35.7–41.1)	47.1 (41.4–49.5)
50–99	50.0 (45.1–56.2)	56.9 (50.6–62.6)	66.4 (58.2–71.9)	80.0 (67.6–86.1)
**AIDS mortality rate when not on ART, deaths per 100 person‐years**				
>500	0.0 (0.0–0.0)	0.0 (0.0–0.0)	0.0 (0.0–0.0)	0.0 (0.0–0.0)
350–500	0.0 (0.0–0.0)	0.0 (0.0–0.0)	0.0 (0.0–0.0)	0.0 (0.0–0.0)
250–349	0.2 (0.1–0.2)	0.2 (0.1–0.3)	0.3 (0.2–0.4)	0.3 (0.2–0.4)
200–249	0.7 (0.5–1.0)	1.0 (0.7–1.3)	1.2 (0.9–1.6)	1.5 (1.0–1.9)
100–199	4.8 (3.8–5.8)	6.4 (5.3–7.6)	8.0 (6.7–9.6)	9.7 (8.0–11.7)
50–99	22.7 (19.0–27.3)	30.5 (27.1–36.5)	38.2 (34.3–46.4)	45.9 (40.6–57.3)
<50	71.2 (60.8–91.8)	95.4 (86.0–121.8)	119.7 (108.4–154.7)	143.9 (128.9–188.1)

HIV‐related mortality rates among 15‐ to 24‐year‐olds decreased 2.3% (95% CrI: 2.1–2.5%) per unit increase in CD4 count (Figure 2b), from a maximum of 0.59 per person‐year (95% CrI: 0.51–0.69) at zero CD4 cells/mm^3^. The 2.03‐fold (95% CrI: 1.83–2.60) mortality rate ratio indicates that estimated HIV‐related mortality rates were about twice the mortality rates among seroconverters observed in the CASCADE Collaboration.

Compared to ages 15–24, age 25–34 at seroconversion was associated with a 4.4% (95% CrI: 3.6–5.5%) lower median initial CD4 count and 13% (95% CrI: 10.4–14.2%) shorter depletion time (Table 2). HIV‐related mortality rates were 34% (95% CrI: 23–50%) higher in 25‐to 34‐year‐old PLHIV compared to 15‐ to 24‐year‐old PLHIV. These effects accrued linearly in successive age groups.

### 3.2 Model fit to training data

Initial CD4 counts were available from 13,176 individuals. The log‐logistic distribution fitted these data closely (Appendix S2). The estimated proportion with 200–349 CD4 cells/mm^3^ was slightly higher than the observed distribution and proportion with 350–500 cells/mm^3^ slightly lower but provided a higher‐likelihood fit than alternate distributions considered (lognormal or gamma).

**Figure 3:**
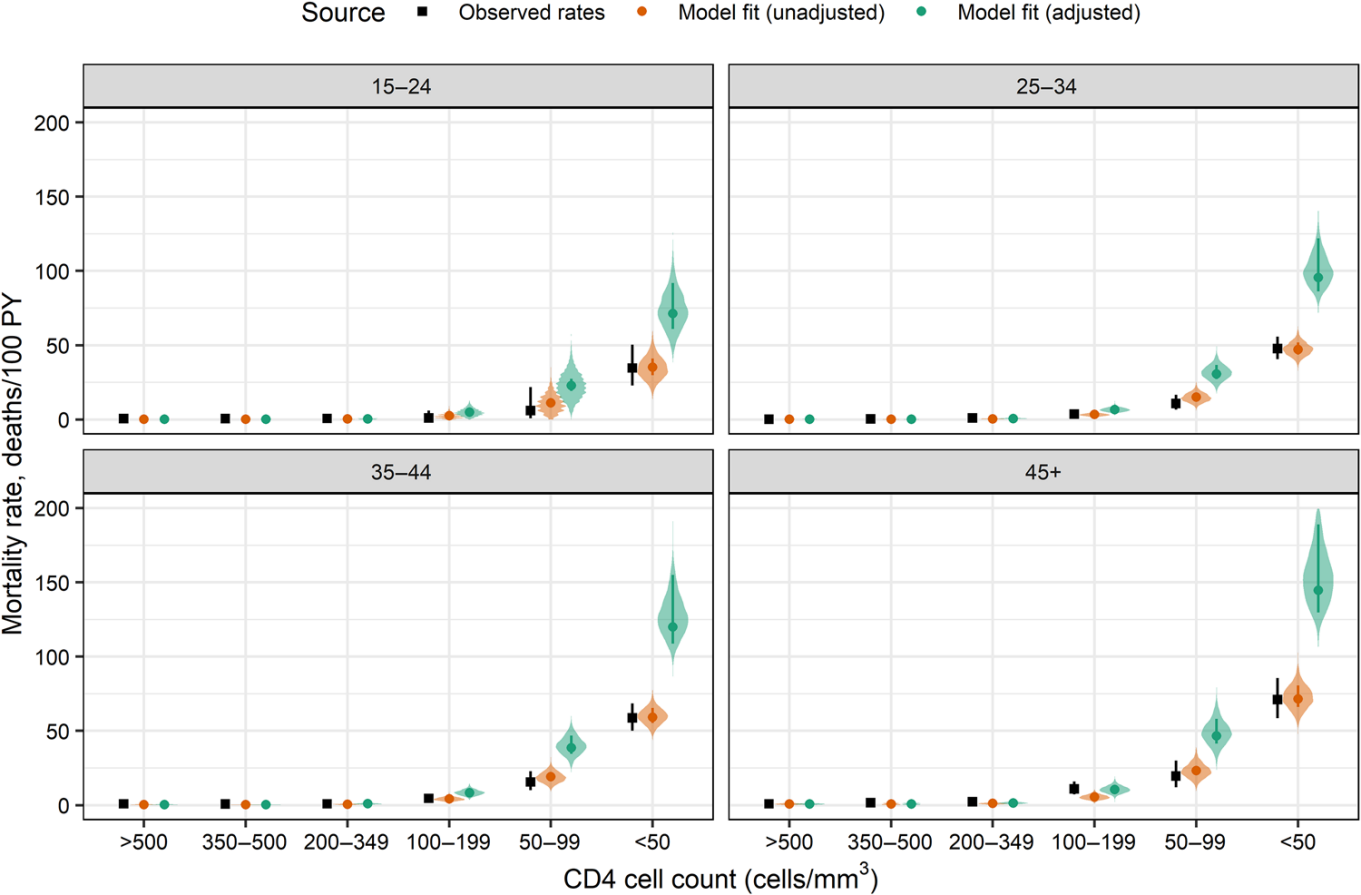
Model fit to all‐cause mortality data from the CASCADE Collaboration reported by Dunn et al. [28]. Unadjusted mortality rates used $\varphi_{4}=\;1$ and were intended to fit observed rates closely. Adjusted rates used estimated values of $\varphi_{4}$ that may account for mortality underestimation in the observed rates. Points show crude observed rates and model point estimates; whiskers show 95% exact Poisson confidence intervals on observed rates and 95% credible intervals on model estimates. Shaded regions show posterior predictive distributions on model estimates. Abbreviation: PY, person‐years.

CASCADE data on mortality by CD4 category consisted of 665 deaths during 12,679 person‐years. Fitted unadjusted mortality rates were close to observed rates in most age groups and CD4 categories (Figure 3). The large credible intervals about the adjusted rates reflect substantial uncertainty in mortality rate and rate ratio estimates.

**Figure 4:**
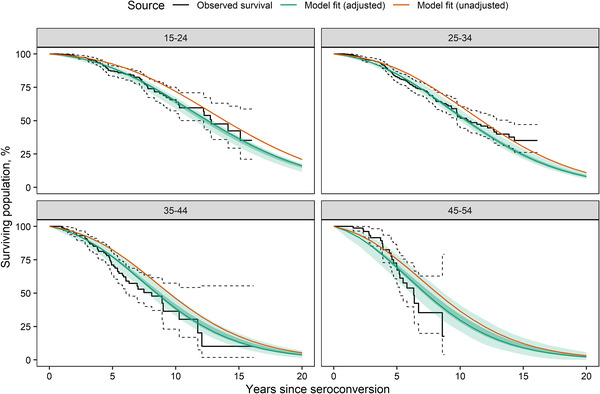
Survival after seroconversion. We calculated Kaplan–Meier survival curves and associated confidence intervals from times from estimated seroconversion date to death or censoring in four East African cohorts [7] (“observed survival”). Solid green curves were calculated using posterior mode natural history parameter values from Table 3. Shaded regions show 95% credible intervals (dark green) and 95% posterior predictive intervals (light green) about model‐based estimates. Solid orange curves were calculated using the posterior mode inputs without mortality rate adjustment ( $\varphi_{4}=1$); uncertainty intervals for unadjusted survival curves are omitted for clarity.

We included survival data for 1421 seroconverters from Tanzania (*n* = 272), Uganda (*n* = 1002) and Rwanda (*n* = 147), of whom 279 died during follow‐up. Fitted median survival times were 12.5 (95% CrI: 12.1–12.7), 10.5 (95% CrI: 10.3–10.7), 8.5 (95% CrI: 8.3–8.8) and 7.2 (95% CrI: 7.1–7.7) years for people aged 15–24, 25–34, 35–44 and 45–54 at seroconversion, respectively. Figure 4 compares our fitted estimates to Kaplan–Meier estimates by age group. Fitted survival curves were consistent with Kaplan–Meier point estimates throughout most of the data period, but the predicted proportion of individuals aged 25–34 at seroconversion who survived at least 12 years (Figure 4) was below crude estimates, and the proportion of 45–54 seroconverters who died within 4 years of seroconversion was above crude estimates.

**Figure 5:**
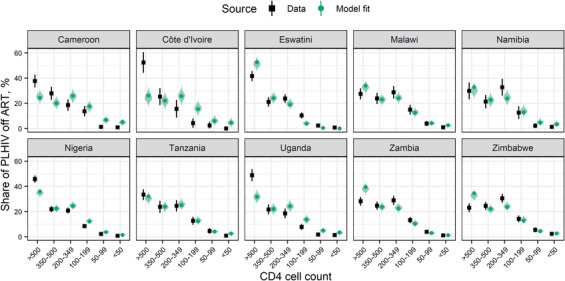
Model fit to CD4 counts measured in PHIA respondents living with HIV off ART. Black points and whiskers show survey‐based point estimates and 95% confidence intervals, respectively. Confidence intervals shown were calculated via jackknife. Green points, whiskers and shaded regions show model point estimates, 95% credible intervals and posterior predictive distributions, respectively. Abbreviations: ART, antiretroviral therapy; PHIA, population‐based HIV impact assessment.

We included CD4 count data measured in 6034 HIV‐positive untreated PHIA respondents. Larger proportions of respondents had CD4 counts above 200 cells/mm^3^, while relatively few had lower CD4 counts. Posterior predictive distributions of modelled proportions contained 63% (38/60) survey‐based point estimates (Figure 5). Modelled proportions with CD4>500 cells/mm^3^ agreed closely with the survey‐based estimates in only two countries: Tanzania and Namibia. Proportions with CD4>500 cells/mm^3^ were underestimated in four countries (Cameroon, Cote d'Ivoire, Nigeria and Uganda) and overestimated in four (Eswatini, Malawi, Zambia and Zimbabwe).

### 3.3 Model validation

**Figure 6:**
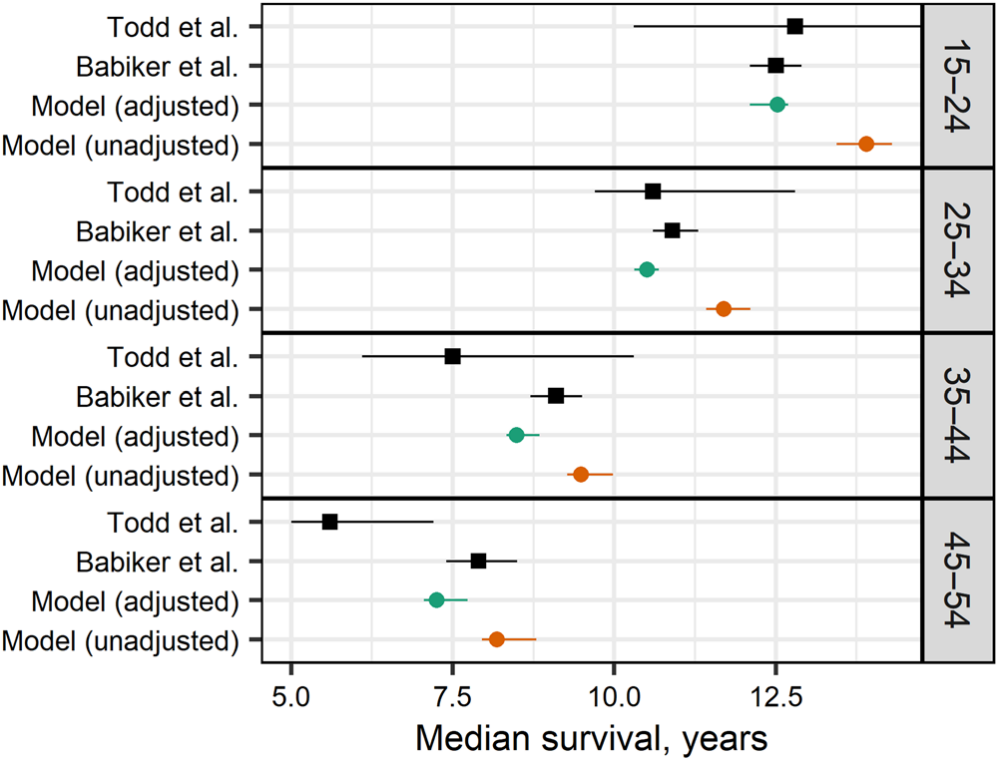
Median survival by age at HIV seroconversion. We compare survival in seroconverter cohorts in East Africa published by Todd et al. [7] and Europe, North America and Australia published by Babikeret al. [32] to adjusted (green) and unadjusted (orange) model estimates. Unadjusted estimates assumed that all‐cause mortality data were unbiased ( $\varphi_{4}=\;1$). Whiskers show 95% confidence intervals on published estimates and 95% credible intervals about model estimates. Note that we compare the estimate from Todd et al. for ages 45 and above to age 45–54 estimates from the other sources shown. Data from Todd et al. were used for model fitting; model estimates shown include background mortality for Uganda contemporary with that study.

Compared to published median survival times in European, North American and Australian seroconverter cohorts, adjusted survival time estimates after seroconversion agreed well at ages 15–34 (Figure 6). Fitted median survival for the adjusted inputs was shorter than observed survival in 35‐ to 54‐year‐old seroconverters from these regions, while median survival for the unadjusted inputs was longer than observed survival in 15‐ to 34‐year‐old seroconverters despite overlapping patient populations in the survival time and mortality rate studies [28, 32].

**Figure 7:**
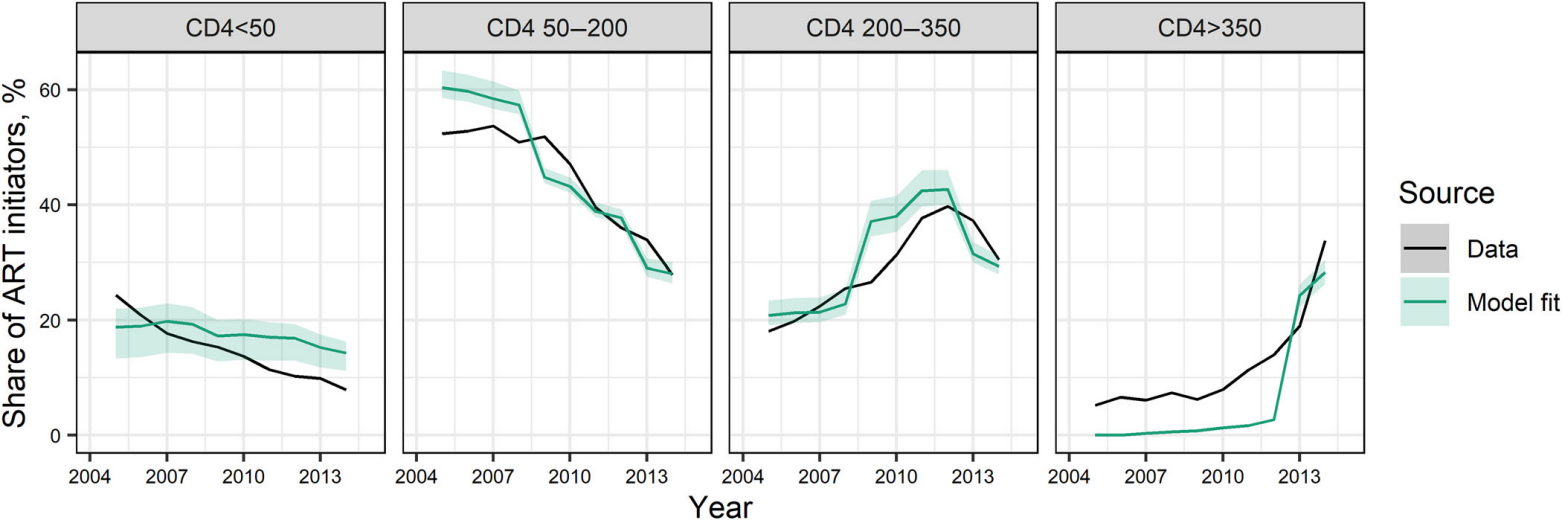
CD4 cell counts at ART initiation. Panels compare the shares of patients starting ART in different CD4 categories. Solid curves show point estimates from IeDEA Consortium data [31] (black) and model estimates (green), shaded areas show 95% posterior predictive intervals about model estimates. Abbreviation: ART, antiretroviral therapy.

We compared CD4 count distributions at ART initiation to fitted model trends (Figure 7). Cohort data were available from 471,220 ART‐naïve patients in seven countries (65% from Zambia; Malawi, 10%; Uganda, 9%; Nigeria, 5%; Côte d'Ivoire, 4%; Zimbabwe, 4%; and Tanzania, 3%). Observed initiation at CD4 counts below 50 cells/mm^3^ declined from 24% in 2005 to 8% in 2014. The modelled proportion was comparable but declined more slowly, from 19% (95% CrI: 13–22%) to 14% (95% CrI: 11–16%). Modelled proportions initiating ART at 50–200 and 200–350 CD4 cells/mm^3^ were similar to observed trends. Spectrum imposes national treatment guidelines, which limited treatment to PLHIV with lower CD4 counts (or members of specific eligible populations like pregnant women or tuberculosis patients) before the World Health Organization raised recommended CD4 count thresholds for ART initiation in 2013 and 2015 [34, 35]. Consequently, modelled proportions starting ART at CD4 counts above 350 cells/mm^3^ (<3%) were substantially lower than observed proportions (5–14%) before 2013.

### 3.4 Sensitivity analysis

Sex‐disaggregated data were available from two training data sources (initial CD4 counts and survival after seroconversion). We considered an alternate model that scaled progression and mortality rates for females by a common rate ratio relative to males. The posterior rate ratio estimate (0.99, 95% CrI: 0.94–1.02) suggested that sex differences were negligible (Table S1).

## 4 DISCUSSION

Since 2015, the World Health Organization has recommended offering ART to adults living with HIV regardless of CD4 cell counts [35]. While the role of CD4 count in managing HIV patient care is diminished, it remains an important prognostic marker for modelling disease progression, AIDS‐related death and care seeking. We used CD4 counts measured in PHIA household survey respondents living with HIV and among HIV seroconverter cohorts to revise the natural history parameters in Spectrum. These data, in conjunction with data collected on HIV survival before widespread ART availability, allowed us to estimate rates of disease progression and HIV‐related mortality more precisely than with the latter data alone.

This revision was motivated by comparison of Spectrum outputs to PHIA data. Previous natural history assumptions in Spectrum [36] generally resulted in overestimating CD4 counts in untreated adults compared to new survey data. Our updated estimates suggest that newly infected people living with HIV are more likely to have lower initial CD4 counts, and progress through infection stages more rapidly, but experience lower HIV‐related mortality rates in most stages, consistent with other analyses of CD4 progression in HIV seroconverter cohort studies [9, 10, 11].

Our natural history parameter estimates may increase HIV‐related deaths in Spectrum. While our mortality rates are generally lower than previously assumed, mortality remains high in advanced infection stages, and people reach these later stages more quickly. This decreased survival times with untreated HIV slightly compared to previous Spectrum inputs. Due to faster progression, people living with HIV may also initiate ART at lower CD4 counts than previously estimated, which will likely increase modelled estimates of HIV‐related deaths since immune status at ART initiation is a key determinant of mortality on ART [37].

Our synthesis included survey data from the current era of widespread ART availability, which posed important inferential challenges. Our parameter estimates depend on modelled ART uptake and retention dynamics [9] in Spectrum. This sensitivity is both a strength and a limitation. Sensitivity to ART uptake provided leverage to estimate an allocation parameter used to model ART initiation, which had little empirical basis previously. Sensitivity to modelled treatment interruption is a limitation, however. Country analysts may enter national treatment retention rates into Spectrum, but these data are often sparse and confounded by silent transfers between care facilities [37, 38, 39, 40].

Our natural history parameter estimates produced CD4 count distributions that generally matched PHIA data well. However, our model did not explain variation in proportions with CD4 counts above 500 cells/mm^3^. Our model was equally likely to underestimate or overestimate proportions in this stage, which could reflect geographic heterogeneity in CD4 count distributions among HIV‐negative adults [41] and in HIV progression, which may owe to environmental, host or viral factors, such as differences in HIV subtype [9, 10, 42, 43, 44, 45, 46]. We have not investigated these here because estimation of country‐level effects risks overfitting.

Our survival estimates were comparable to previously published survival distributions in East African cohorts and cohorts in Europe, North America and Australia [7, 32]. Previous analyses of longitudinal individual‐level data found that CD4 counts at seroconversion were lower, and progression and mortality rates were higher in southeastern and eastern Asia compared to Europe and North America [9], consistent with relatively short survival times estimated in Thai cohorts [7, 47] and comparative analyses of CD4 count trends between seroconverters in Beijing and the CASCADE Collaboration [48]. Regional variations in natural history assumptions have not been implemented in previous Spectrum versions or our analysis, but could be considered in future multi‐parameter evidence synthesis exercises.

Spectrum previously assumed that disease progression and mortality rates did not vary by sex. We considered relaxing this assumption, but our estimated sex differences were negligible. This is consistent with previous analyses, which suggest that sex differences may be small [7, 9, 11, 46] after adjusting for age and immune status. Nevertheless, we regard our finding with caution. Our study had limited power to identify sex differences because only two data sources were disaggregated by sex.

Our analysis has several limitations. Our synthesis used data from sub‐Saharan Africa, Europe, North America and Australia, and may not generalize to other epidemic contexts. Our mortality rate estimates by CD4 category were about twice as large as observed among seroconverters in the CASCADE Collaboration [28]. This may indicate bias in the data on mortality by CD4 category, for example, potentially due to better management of opportunistic infections, but we were unable to determine specific mechanisms for that apparent bias. We used national HIV epidemic models to calculate CD4 count distributions for comparison to survey and ART initiator data, thus our natural history parameter estimates may be sensitive to other structural or input assumptions in those models.

## 5 CONCLUSIONS

Our update to natural history parameter estimates formally synthesizes information from multiple, important sources of information on HIV disease progression and mortality. New data on immune status of HIV‐positive PHIA respondents provide the power necessary to identify specific parameters, adjust for potential biases and strengthen the empirical basis for the timing of ART initiation in Spectrum.

## FUNDING

Work by RG, SGM and JS was funded by a grant from the Bill & Melinda Gates Foundation (OPP 1191665). The PHIA project was supported by the U.S. President's Emergency Plan for AIDS Relief (PEPFAR) through CDC under the terms of cooperative agreement #U2GGH001226 and #U2GGH001271 (SHIMS2). JWE was supported by UNAIDS, the Bill and Melinda Gates Foundation (OPP1190661), National Institute of Allergy and Infectious Disease of the National Institutes of Health under award number R01AI136664 and the MRC Centre for Global Infectious Disease Analysis (reference MR/R015600/1), jointly funded by the UK Medical Research Council (MRC) and the UK Foreign, Commonwealth & Development Office (FCDO), under the MRC/FCDO Concordat agreement and is also part of the EDCTP2 programme supported by the European Union.

## DISCLAIMER

The findings and conclusions in this report are those of the authors and do not necessarily represent the official position of the funding agencies.

## COMPETING INTERESTS

The authors declare that they have no competing interests.

## AUTHORS’ CONTRIBUTIONS

RG, JS and JWE conceptualized this study. SJ, NK, SB, LFJ, TM, NP and JWE contributed to data collection. RG, SJ, NK and JS analysed the data. RG, SGM, LFJ, TB, JS and JWE interpreted the results. RG wrote the first draft of the manuscript, which was revised by all authors. All authors have reviewed and approved the final version of the manuscript.

## Supporting information

**Table S1**. Model sensitivity to sex differences in progression and mortality.
