# Supplementary material for: Disease progression and mortality with untreated HIV infection: evidence synthesis of HIV seroconverter cohorts, antiretroviral treatment clinical cohorts and population‐based survey data
Source: J Int AIDS Soc. 2021 Sep 21;24(Suppl 5):e25784. doi: 10.1002/jia2.25784 (PMC8454684; doi:10.1002/jia2.25784)
Supplement: Supplementary file 1 [file JIA2-24-e25784-s002.docx]

Appendix S1: Detailed description of methods to “**Disease progression and mortality with untreated HIV infection: evidence synthesis of HIV seroconverter cohorts, antiretroviral treatment clinical cohorts, and population-based survey data**”

# Overview

This appendix provides a detailed description of our continuous natural history model (Section 2) and the mapping from continuous model parameters to discrete Spectrum inputs (Section 3). Section 4 describes the observational models used specify the likelihood of training data. Section 5 discusses our use of validation data. Section 6 describes estimation of posterior predictive distributions. Figure S1 illustrates the dependencies between natural history model parameters, Spectrum inputs, and training data. Figure S2 compares prior and posterior distributions of continuous natural history inputs.


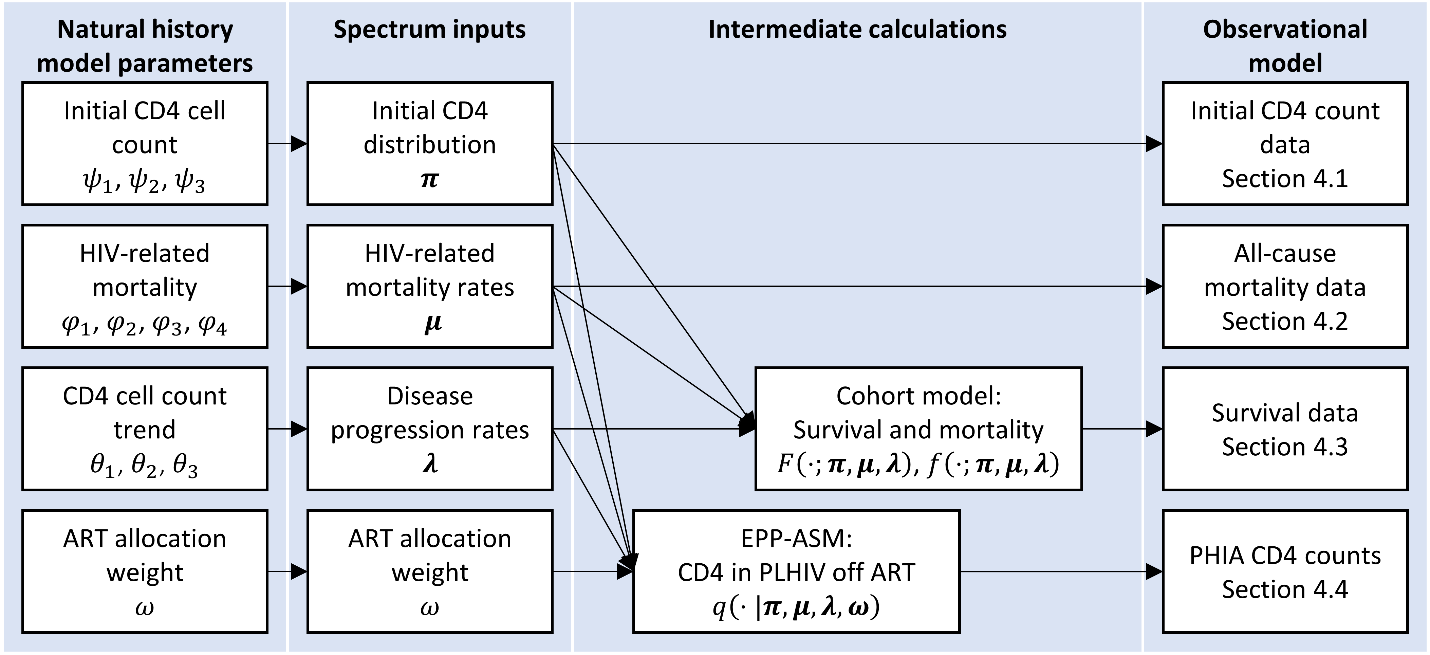


**Figure S1. Influence diagram.** This diagram shows the relationship between natural history model parameters, Spectrum inputs, and observational models used to derive the likelihood of training data. We assumed input initial CD4 distributions and HIV-related mortality rates directly influenced corresponding data on initial CD4 counts and all-cause mortality, respectively, while survival and cross-sectional CD4 count data depended on intermediate calculations done using models derived from Spectrum.

We implemented our evidence synthesis in R [[1](#_ENREF_1)]. We used an implementation of IMIS (available from <https://cran.r-project.org/src/contrib/Archive/IMIS/>) that we modified to operate on log-transformed probability densities to improve numerical stability. We implemented a cohort model in C++ callable from R to evaluate the likelihood of survival data given natural history model parameter estimates; similarly, we used the EPP-ASM HIV epidemic model available in the first90 package for R [[2](#_ENREF_2)] to calculate outputs, cross-sectional CD4 counts in adult PLHIV off ART and at ART initiation, conditional on natural history parameter values. We used these outputs to evaluate the likelihood of CD4 counts measured in untreated PHIA respondents with HIV, and as a comparator to ART initiator cohort data used for model validation, respectively. The R and C++ code we used is available online at <https://github.com/rlglaubius/NaturalHistorySynthesis>. Most datasets included in our synthesis are confidential and could not be shared. The Spectrum software is freely available for use on Windows-based computers, and can be downloaded from <https://avenirhealth.org/software-spectrum.php> or accessed online at <https://aim.spectrumweb.org>.

# Natural history model

Spectrum requires 80 input values to fully specify HIV natural history (main text Figure 1). However, since these inputs may be correlated when differences in age or CD4 cell count are small, the number of degrees of freedom needed to represent those inputs may be smaller. We used a parsimonious, eleven parameter model to estimate Spectrum’s natural history inputs. We modelled continuous initial CD4 cell count distributions, continuous CD4 count declines after HIV seroconversion, and HIV-related mortality rates that vary smoothly with CD4 counts. These parameters are detailed below and summarized in main text Table 2. In the following, subscripts $a$ = 0, 1, 2 or 3 denote age groups 15-24, 25-34, 35-44 or 45+, respectively.

## Initial CD4 cell counts

We modeled initial CD4 cell counts using a log-logistic distribution with age-specific median $m_{a}$ and shape $\psi_{1}$. We specified the median relative to ages 15-24 ($m_{0}=\psi_{2}$) via a linear age effect ($\psi_{3}$). The proportion of seroconverters with initial CD4 cell counts of $c$ or less is as follows.

$P_{a}\left( C\leq c \right)=1/\left[ 1+\left( \frac{c}{m_{a}} \right)^{-\left( \psi_{1}+1 \right)} \right]$ with $m_{a}=\psi_{2}\cdot\left( 1-\psi_{3}a \right)$ (1)

We used a diffuse prior $\psi_{1}\sim\mathrm{Exponential}(1/3)$ to admit modes well above zero CD4 cells/mm^3^. We assumed $\psi_{2}\sim Gamma(\sqrt{585}, \sqrt{585})$ since 15-24-year-old seroconverters have initial CD4 counts near 585 cells/mm^3^ on average [[3](#_ENREF_3), [4](#_ENREF_4)], and $\psi_{3}\sim Uniform(0, 1/3)$ because initial CD4 cell counts may fall with increasing age at seroconversion [[3](#_ENREF_3), [5](#_ENREF_5)].

## CD4 trends after seroconversion

We modeled CD4 cell counts as decreasing continuously over years $t$ since seroconversion according to polynomial curves. We specified these curves $c_{a}\left( t \right)$ in terms of a baseline CD4 cell count $c_{a}\left( 0 \right)$, an age-specific “depletion time” $\tau_{a}$ when CD4 counts reach zero cells/mm^3^, and shape parameter $\theta_{1}$. We specified depletion times relative to ages 15-24 ($\tau_{0}=\theta_{2}$) via a linear age effect ($\theta_{3}$).

$c_{a}\left( t \right)=c_{a}\left( 0 \right)\left( 1-\frac{t}{\tau_{a}} \right)^{\theta_{1}+1}$ for $\tau_{a}=\theta_{2}\cdot\left( 1-\theta_{3}a \right)$ (2)

We calculated baseline CD4 cell counts $c_{a}\left( 0 \right)$ as the median among seroconverters with initial CD4 counts over 500 cells/mm^3^ (Equation 1).

Our $\theta_{1}\sim\mathrm{Exponential}\left( 1 \right)$ prior produces quadratic trends on average, consistent with previous modeling of longitudinal CD4 count measurements [[6](#_ENREF_6)]. Since most untreated PLHIV die before reaching zero CD4 cells/mm^3^ [[7](#_ENREF_7)], we have no direct evidence to inform the depletion time prior. We formulated this prior, $\theta_{2}\sim\mathrm{Lognormal}(3, 0.29)$, to have mode 15 years and median 20 years to admit depletion times comparable to, but longer than, estimated survival among 15-24-year-old seroconverters [[5](#_ENREF_5), [8](#_ENREF_8), [9](#_ENREF_9)]. We assume CD4 cell counts may decline more rapidly with age, $\theta_{3}\sim\mathrm{Uniform}(0, 1/3)$ [[10](#_ENREF_10), [11](#_ENREF_11)].

## Mortality by CD4 cell count

We modeled HIV-related mortality rates $\mu_{a}\left( c \right)$ that increase rapidly as CD4 cell counts $c$ decline [[12](#_ENREF_12)].

$\mu_{a}\left( c \right)=\varphi_{1}^{c}\cdot\varphi_{2}\cdot\left( 1+\varphi_{3}a \right)\cdot\varphi_{4}$ (3)

The shape parameter $\varphi_{1}$ controls how rapidly mortality increases as CD4 cell counts fall. Other parameters specify mortality rates among 15-24-year-olds with zero CD4 cells/mm^3^ ($\varphi_{2}$), a linear age effect ($\varphi_{3}$), and a mortality rate ratio ($\varphi_{4}$) that adjusts for potential bias in CASCADE all-cause mortality training data.

Equation 3 was previously used to derive mortality inputs for the Thembisa model [[12](#_ENREF_12)]. Estimates of $\varphi_{1}$ were consistently near 0.988, while estimates of $\varphi_{2}$ varied more across data sets [[12](#_ENREF_12)]. Therefore we used an informative prior $\varphi_{1}\sim\mathrm{Beta}\left( 252, 4 \right)$ concentrated near 0.988, and a weaker prior $\varphi_{2}\sim\mathrm{Gamma}(3.8, 0.25)$ that has mode near Thembisa’s value of 0.645. We assumed HIV-related mortality rates may increase with age, $\varphi_{3}\sim\mathrm{Exponential}\left( 1 \right)$. We imposed a $\varphi_{4}\sim\mathrm{Gamma}(2,1)$ prior that admits upward or downward bias in all-cause mortality data but has mode 1 (no evidence of bias).

## Treatment initiation

We assumed a non-informative $\omega\sim\mathrm{Uniform}\left( 0,1 \right)$ prior on Spectrum’s treatment allocation weight.

# Spectrum input calculation

The mapping from the continuous natural history model to Spectrum’s discrete infection stages (CD4>500, 350-500, 250-349, 200-249, 100-199, 50-99, <50cells/mm^3^; main text Figure 1) depends on the lower and upper CD4 count limits for each stage. We denote the lower limit for stage $h$ by $c_{h,0}$ and the upper limit by $c_{h,1}$. We use $c_{h,0}=0$ cells/mm^3^ for the open-ended CD4<50 cells/mm^3^ stage. We use different values for $c_{h,1}$ in the CD4>500 cells/mm^3^ stage when calculating different Spectrum inputs. We use $c_{h,1}=\infty$ for the initial CD4 cell count distribution so that probabilities $\pi_{h,a}$ sum to one, but use finite values of $c_{h,1}$ when calculating progression and mortality rates to ensure those rates are well-defined.

## Initial CD4 cell counts

Spectrum’s initial CD4 cell count inputs $\pi_{h,a}$ can be calculated directly from the modeled distribution $P_{a}$ for each stage $h$:

$\pi_{h,a}=P_{a}\left( c_{h,0}\leq C<c_{h,1} \right)$ (4)

## Disease progression rates

Since $c_{a}\left( t \right)$ is the modeled CD4 cell count $t$ years after seroconversion, its inverse $c_{a}^{-1}\left( x \right)$ is the number of years after seroconversion when CD4 cell count $c_{a}\left( t \right)=x$,

$c_{a}^{-1}\left( x \right)=\tau_{a}\left( 1-\left[ \frac{x}{c_{a}\left( 0 \right)} \right]^{\frac{1}{\theta_{1}+1}} \right)$ (5)

Spectrum’s progression rates are the reciprocal of the time between entering and exiting stage $h$:

$\lambda_{h,a}=\left[ c_{a}^{-1}\left( c_{h,0} \right)-c_{a}^{-1}\left( c_{h,1} \right) \right]^{-1}$ (6)

When we calculate $\lambda_{h,a}$ for the CD4>500 cells/mm^3^ stage, we set $c_{h,1}$ to the median initial CD4 cell count among people whose initial CD4 cell count is above 500 cells/mm^3^. We calculate $c_{h,1}$ separately for each age group since our modeled initial CD4 distributions may vary by age.

## HIV-related mortality rates

We calculate HIV-related mortality rates for Spectrum $\mu_{h,a}$ as the average mortality rate $\mu_{a}\left( c \right)$ among CD4 cell counts in stage $h$:

$\mu_{h,a}=\frac{1}{c_{h,1}-c_{h,0}}\int_{c_{h,0}}^{c_{h,1}} \mu_{a}\left( x \right)dx$ (7)

Since $\mu_{a}\left( c \right)$ is near zero for $c\geq500$ cells/mm^3^, the choice of $c_{h,1}$ is negligible for this stage. For simplicity, we used $c_{h,1}=685$ cells/mm^3^.

# Observational model

We included four training data sources in our synthesis. We assume these data sources are conditionally independent given the model parameters, which implies their joint likelihood is the product of the marginal likelihood of each data source. We describe the observational models used to derive these marginal likelihoods below.

## Initial CD4 cell counts

To inform initial CD4 cell counts, we included data on CD4 cell counts measured within one year of seroconversion compiled from 13,176 seroconverters in Europe, North America, Australia, and Sub-Saharan Africa between 1982 and 2012 [[5](#_ENREF_5)]. Data consisted of seroconverter counts tabulated by sex, CD4 category (CD4≥500, 350-499, 200-349, 100-199, 50-99, <50 cells/mm^3^), and five-year age group (15-19 to 55-59) at seroconversion. We aggregated seroconverter counts across sex to ten-year age groups (15-24, 25-34, 35-44, 45+). We derived the likelihood model for these data by assuming the aggregated counts $N_{j,a}$ by CD4 category $j$ and ten-year age group $a$ were multinomially distributed given the initial CD4 distribution $\pi_{h,a}$,

$\left( N_{j,a} \right)_{j=1}^{6}\sim\mathrm{Multinomial}\left( N_{a},\left( \pi_{1,a},\pi_{2,a},\pi_{3,a}+\pi_{4,a},\pi_{5,a},\pi_{6,a},\pi_{7,a} \right) \right)$ where $N_{a}=\sum_{j=1}^{6} N_{a,j}$ (8)

We assume the data for each age group are conditionally independent given the model parameters. The seroconverter dataset aggregates two Spectrum infection stages (CD4 200-249 and 250-349 cells/mm^3^) into a single stage, so we add the probabilities of these two stages ($\pi_{3,a}+\pi_{4,a}$) when calculating the likelihood. We use index $j$ (instead of $h$) to distinguish the six CD4 categories in the data from the seven Spectrum stages.

## All-cause mortality rates by CD4 category

To inform HIV-related mortality rates by CD4 category, we included all-cause mortality data from the CASCADE Collaboration [[7](#_ENREF_7)] consisting of 12,679 person-years of follow-up and deaths observed in 1997-2004 among untreated HIV seroconverters by age and infection stage. These data were extracted from Table 2 of Reference [[7](#_ENREF_7)] and consist of numbers of deaths and person-years of observation by age (15-24, 25-34, 35-44, 45-54, 55+) and CD4 category (CD4≥500, 350-499, 200-349, 100-199, 50-99, <50 cells/mm^3^). We aggregated the 45-54 and 55+ age groups to align with Spectrum’s 45+ age group, then derived the likelihood model by assuming the number of deaths $D_{j,a}$ by CD4 category $j$ and age group $a$ were Poisson-distributed given the number of person-years of observation $Y_{j,a}$ and the sum of modeled HIV-related mortality rates $\mu_{j,a}$ and age-specific background mortality rates $\nu_{a}$,

$D_{j,a}\sim\mathrm{Poisson}\left( Y_{j,a}\cdot\left[ \mu_{j,a}/\varphi_{4}+\nu_{a} \right] \right)$ (9)

Note that the mortality rate ratio $\varphi_{4}$ is intended to adjust for potential differences in mortality in this study in comparison to other settings (e.g. if patients in the study received an unusually high standard of care), or study designs (e.g., some individuals may have progressed to later infection stages between their last CD4 count measurement and death). Therefore, we use unadjusted mortality rates (by factoring out $\varphi_{4}$) to evaluate the likelihood of these data. We assume the data for each age group and CD4 category are conditionally independent given the model parameters. Like the seroconverter data above, the all-cause mortality dataset aggregated 200-249 and 250-349 CD4 cells/mm^3^ stages into one CD4 category. We calculate the HIV-related mortality rate for this CD4 category as the weighted average of the mortality rates in the constituent Spectrum stages $(2\mu_{3,a}+\mu_{4,a})/3$, which is equivalent to the corresponding average calculation from our continuous HIV-related mortality model, $\int_{200}^{350} \mu_{a}\left( x \right)dx/(350-200)$.

Since this dataset recorded all deaths, not just HIV-related deaths, we included background mortality rates $\nu_{a}$ in the likelihood. We used a sex-weighted average of all-cause mortality rates among adult males (86%) and females (14%) in 1985-1990 Europe [[13](#_ENREF_13)] to approximate $\nu_{a}$, since most participating cohorts were European, 86% of patients were male, and first CD4 cell counts were measured during 1983-1995. We used all-cause mortality rates for $\nu_{a}$ because HIV-deducted mortality rates were not available.

## Survival after HIV seroconversion

For overall HIV survival, we used individual-level data from four population-based cohort studies of HIV seroconverters from East Africa [[8](#_ENREF_8)]. Individuals were followed during 1994-2004. Seroconversion dates were identified as the midpoint between last negative and first positive HIV tests done at most four years apart. Individuals exited at death or were censored at end of study or when last known alive. We included data from 1,421 participants aged 15-59 at seroconversion. The dataset consisted of individual-level data on study site (Kisesa, Tanzania; Masaka, Uganda; Rakai, Uganda; Rwanda maternal and child health clinic), participant sex, date of birth, HIV seroconversion date, time of study exit, and whether exit was due to death versus censoring. We used dates of birth and seroconversion to calculate age at seroconversion. Since survival after seroconversion in the pre-ART era depended on underlying non-HIV mortality, initial CD4 cell counts, and rates of disease progression and HIV-related mortality, we anticipated that this dataset would inform the latter three components of our natural history model.

Our continuous natural history model does not include aging or background mortality, so it cannot be used to calculate the likelihood of survival data directly. Therefore, we implemented a compartmental cohort model that replicates Spectrum natural history inputs and implements its adult HIV natural history (main text Figure 1), aging, and background mortality components. This cohort model excludes Spectrum dynamics of ART uptake and interruption since these cohort studies were conducted before ART was available in East Africa. We used this cohort model to calculate quantities needed to evaluate the likelihood of the survival data, described below.

We denote by $\boldsymbol{\pi}$ the matrix of Spectrum inputs $\pi_{h,a}$ for stages $h\in\left\{ 1,\ldots7 \right\}$ and ten-year age groups $a\in$ {15-24, 25-34, 35-44, 45+}. Similarly, we let $\boldsymbol{\mu}$ and $\boldsymbol{\lambda}$ denote matrices of Spectrum HIV-related mortality and disease progression inputs. Using this notation, the cohort model has three key outputs: The survival function $F(t,s,b;\boldsymbol{\pi},\boldsymbol{\mu},\boldsymbol{\lambda})$ quantifies the proportion of people by sex $s$ who seroconvert at age $b\in\{15, 16, 17,\ldots\}$ who are still alive $t$ years after seroconversion given inputs $\boldsymbol{\pi},\boldsymbol{\mu},\boldsymbol{\lambda}$. The stage occupancy function $G\left( h,t,s,b;\boldsymbol{\pi}, \boldsymbol{\mu}, \boldsymbol{\lambda} \right)$ quantifies the proportion of these survivors who are in infection stage $h$. Finally, the mortality density function $f\left( t,s,b;\boldsymbol{\pi}, \boldsymbol{\mu}, \boldsymbol{\lambda} \right)$ is the product of the survival function and the mortality hazard among survivors:

$f\left( t,s,b;\boldsymbol{\pi}, \boldsymbol{\mu}, \boldsymbol{\lambda} \right)=F\left( t,s,b;\boldsymbol{\pi}, \boldsymbol{\mu}, \boldsymbol{\lambda} \right)\left( \nu_{s,b+t}\left( t_{0}+t \right)+\sum_{h=1}^{7} G\left( h,t,s,b;\boldsymbol{\pi}, \boldsymbol{\mu}, \boldsymbol{\lambda} \right)\mu_{h,b+t} \right)$ (10)

The mortality hazard depends on sex-specific background mortality rates $\nu_{s,b+t}\left( t_{0}+t \right)$ and HIV-related mortality rates $\mu_{h,b+t}$ (We abuse notation slightly to let $\mu_{h,b+t}$ be the input HIV-related mortality rate for the ten-year age group that includes exact age $b+t$). Since the survival dataset does not include CD4 cell count measurements, we average the mortality hazard across HIV infection stages based on the stage occupancy function $G\left( h,t,s,b;\boldsymbol{\pi}, \boldsymbol{\mu}, \boldsymbol{\lambda} \right)$. Since 70% of seroconverters were from Ugandan cohorts, we approximated background mortality rates $\nu_{s,b+t}\left( t_{0}+t \right)$ by all-cause mortality rates for Uganda [[13](#_ENREF_13), [14](#_ENREF_14)] by sex $s$ and exact age $b+t$ relative to reference year $t_{0}=1997$.

We use the above cohort model outputs to calculate the survival likelihood for right-censored data [[15](#_ENREF_15)]. Let $t_{i}$ be the observed survival time, $\delta_{i}$ the censoring indicator ($\delta_{i}=1$ if death observed, $\delta_{i}=0$ otherwise), $s_{i}$ sex, and $b_{i}$ age at seroconversion for each study participant $i\in\left\{ 1,\ldots,n \right\}$. Then the likelihood of the survival data is

$P(\left\{ \left( t_{i},\delta_{i}) :i=1,\ldots,n \right\} | \boldsymbol{\pi}, \boldsymbol{\mu}, \boldsymbol{\lambda} \right)\boldsymbol{=}\prod_{i:\delta_{i}=1} f(t_{i},s_{i},b_{i};\boldsymbol{\pi}, \boldsymbol{\mu}, \boldsymbol{\lambda)}\prod_{i:\delta_{i}=0} F\left( t_{i},s_{i},b_{i};\boldsymbol{\pi}, \boldsymbol{\mu}, \boldsymbol{\lambda} \right)$ (11)

We weighted the log-likelihood of survival data by a factor of ten, i.e., the number of countries we had cross-sectional household survey data for, so that the contribution of survival data to the joint likelihood would be comparable.

## Cross-sectional CD4 cell counts in survey respondents

We included data from ten Africa-based PHIAs conducted in 2015-2018 (main text Table 1). Data consisted of survey-based estimates of nationally representative CD4 category distributions (CD4≥500, 350-499, 200-349, 100-199, 50-99, <50 cells/mm^3^) in untreated PLHIV by five-year age group (15-19 to 45-49). HIV-positive survey respondents were classified as on ART if they reported ART use or had detectable antiretroviral drugs in blood, and off ART otherwise. The measured CD4 cell counts among PHIA respondents off ART reflect past HIV incidence, HIV natural history, and the effects of ART scale-up. Therefore, we anticipated these data would inform all parameters of our natural history model.

The PHIA data represent the cumulative effects of HIV disease progression and mortality among people who acquired HIV over the years before those surveys. Moreover, some potential respondents may have been “missing” because they started treatment, and some respondents may have previously taken ART. Since our natural history model does not incorporate HIV incidence or the full dynamics of ART scale-up, it does not produce outputs we can compare to the survey data directly. Instead, we used the natural history model to calculate Spectrum inputs, then used an HIV epidemic model, EPP-ASM, to calculate the corresponding distributions of CD4 counts in untreated adults living with HIV. EPP-ASM is designed to closely replicate Spectrum’s adults demographic, epidemiological, and treatment program calculations, and interoperates with files produced using Spectrum [[16](#_ENREF_16)]. These files contain all Spectrum inputs countries used to produce their official HIV estimates, such as HIV incidence trends and numbers on ART. Crucially for purposes of Bayesian inference, EPP-ASM is fast to calculate and is accessible in R [[2](#_ENREF_2)]. We used national Spectrum files derived from the 2019 round of HIV estimates [[17](#_ENREF_17)] to specify all inputs outside our natural history model. EPP-ASM aggregates CD4 reporting across ages 50 and up; we excluded PHIA data for ages 50-54 and older since EPP-ASM does not provide directly comparable outputs.

The PHIA CD4 cell count dataset consisted of numbers of survey respondents living with untreated HIV infection $n_{a,r}$ by country $r$ and five-year age group $a$ (15-19 to 45-49) and the estimated proportion $p\left( j | a,r \right)$ of those respondents in CD4 category $j$ (CD4≥500, 350-499, 200-349, 100-199, 50-99, <50 cells/mm^3^) conditioned on age and country. We derived the likelihood from a multinomial observation model,

$\left( n_{a,r}\cdot p\left( j | a,r \right) \right)_{j=1}^{6}\sim\mathrm{Multinomial}\left( n_{a,r}, q\left( j=1 | a,r,\boldsymbol{\pi},\boldsymbol{\mu,\lambda,}\omega\right),\ldots,q\left( j=6 | a,r,\boldsymbol{\pi},\boldsymbol{\mu,\lambda,}\omega\right) \right)$ (12)

Here, we let $q\left( j | a,r,\boldsymbol{\pi,\mu,\lambda},\omega\right)$ denote the proportion of untreated PLHIV in CD4 category $j$ for age group $a$ and country $r$ in the year the PHIA was conducted, as calculated by EPP-ASM using natural history inputs $\boldsymbol{\pi}$, $\boldsymbol{\mu}$, $\boldsymbol{\lambda}$, and $\omega$.

The PHIA estimates $p\left( j | a,r \right)$ used in our synthesis were calculated from survey data weighted to account for differential selection probabilities, with adjustments for non-response and under coverage of the population by age and sex in each country. Estimates for our analysis used biomarker weights since the analysis was restricted to respondents who participated in the biomarker component of the PHIA survey. Confidence intervals shown in main text Figure 5 were calculated using jackknife replicate weights. Analyses of PHIA data were conducted using SAS survey procedures (v9.4, SAS institute).

# Validation data

We held out two data sources for model validation. We compared our estimated survival times to median survival times estimated in European, Australian, and North American seroconverters [[9](#_ENREF_9)]. We also compared modeled CD4 cell counts at ART initiation to data from ART initiators in IeDEA (International epidemiology Databases to Evaluate AIDS) collaborating clinical cohorts [[18](#_ENREF_18)]. We reserved the survival data for validation, as opposed to formally incorporating them in our evidence synthesis, because only the published median survival times were available.

We held the ART initiator cohort data out of the evidence synthesis for concerns that their inclusion might unduly bias parameter estimates. A substantial proportion of ART initiators had missing CD4 counts, and it is unclear to what extent these missing CD4 data might bias results. Cohort sizes varied substantially across countries [[18](#_ENREF_18)], which could bias parameter estimates towards countries with larger cohorts. Further, collaborating cohorts are not nationally representative. We addressed these in our graphical comparison by pooling cohorts and weighting model-based comparators proportional to cohort sizes in each country, as described in the main text. As with cross-sectional CD4 cell counts (Section 4.4), we compared ART initiator cohort data to contemporary CD4 cell counts at ART initiation calculated using EPP-ASM from our natural history input estimates. However, this comparison may be biased because ART initiator cohorts excluded people who restarted ART after treatment interruption when this could be ascertained, whereas EPP-ASM does not distinguish new initiators from restarts.

# Posterior predictive distributions

We compared posterior predictive distributions (PPD) about model parameters and outputs to training and validation data [[19](#_ENREF_19)]. Whereas credible intervals quantify uncertainty in our natural history parameter estimates, PPDs incorporate both parameter uncertainty and uncertainty from sampling error implicit in the observational models used to specify the likelihood for each data source (Section 4). We generated synthetic datasets by drawing random samples from the observational model for each training data source. We generated one synthetic dataset for each of the 3,000 sets of natural history parameter values obtained from IMIS. PPDs calculated from these synthetic datasets summarized quantities of interest (e.g., mortality rates for a given age group and CD4 category) for comparison to quantities calculated from the corresponding training data. We estimated 95% posterior predictive intervals as the 95% quantile range of PPDs.

Main text Figure 4 compares posterior predictive distributions of survival times to Kaplan-Meier curves estimated from East African seroconverter cohorts. Given a set of natural history parameter values $\boldsymbol{\pi}, \boldsymbol{\lambda},$ and $\boldsymbol{\mu}$, we first calculated corresponding survival functions $F\left( \cdot,s,b;\boldsymbol{\pi},\boldsymbol{\lambda},\boldsymbol{\mu} \right)$ by sex $s$ and single age $b$ (Section 4.3). Next, we drew synthetic, uncensored survival times according to $F$ for each individual in the seroconverter dataset; we used these synthetic survival times to calculate Kaplan-Meier curves by ten-year age group. For Figure 4, we estimated posterior predictive intervals pointwise over time since seroconversion as 95% quantile ranges calculated across Kaplan-Meier curves for each synthetic dataset. We assumed all survival times were known in the synthetic datasets; our posterior predictive intervals would have been wider if we had modeled censoring.

In addition to PPDs on training data, we estimated PPDs for validation data on CD4 counts at ART initiation (main text Figure 7). We generated synthetic ART initiator data sets by assuming the distribution of CD4 categories at ART initiation was multinomially-distributed, conditional on observed numbers starting ART by country, year, and sex, and conditioned on the distribution of CD4 categories at ART initiation calculated using EPP-ASM from posterior natural history parameter estimates. Due to the large numbers of ART initiators, posterior predictive intervals and credible intervals were almost identical.


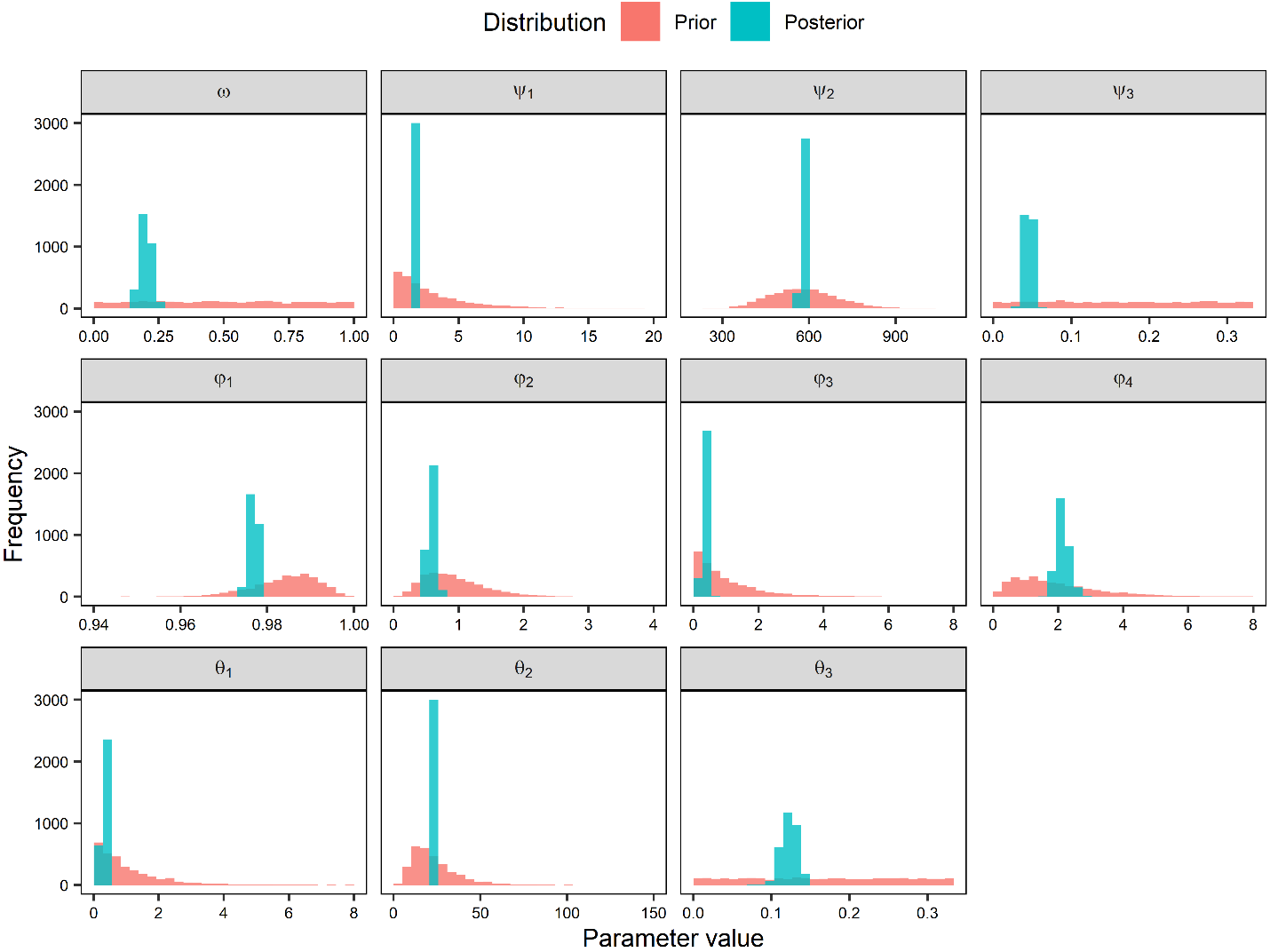


**Figure S2. Comparison of prior and posterior distributions on natural history model parameters.** Histograms in each panel compare 3,000 prior distribution samples to 3,000 posterior samples. Parameters specify the ART allocation weight ($\omega$), initial CD4 cell count distribution ($\psi$), HIV-related mortality rates ($\varphi$), and CD4 cell count declines ($\theta$) as described in main text Table 2.

**REFERENCES**

1. R Development Core Team. R: A language and environment for statistical computing. Vienna, Austria: the R Foundation for Statistical Computing; 2011.

2. Eaton J, Maheu-Giroux M, Giguère K, Bhatkoti R. first90: The first90 model 2020. Available from: <https://github.com/mrc-ide/first90release>.

3. Lodi S, Phillips A, Touloumi G, Geskus R, Meyer L, Thiébaut R, et al. Time from human immunodeficiency virus seroconversion to reaching CD4+ cell count thresholds <200, <350, and <500 cells/mm^3^: assessment of need following changes in treatment guidelines. Clin Infect Dis. 2011;53(8):817-25. doi: 10.1093/cid/cir494.

4. Touloumi G, Pantazis N, Pillay D, Paraskevis D, Chaix M-L, Bucher HC, et al. Impact of HIV-1 subtype on CD4 count at HIV seroconversion, rate of decline, and viral load set point in European seroconverter cohorts. Clin Infect Dis. 2013;56(6):888-97. doi: 10.1093/cid/cis1000.

5. Mangal TD, the UNAIDS Working Group on CD4 Progression and Mortality Among Seroconverters, the CASCADE Collaboration in EuroCoord. Joint estimation of CD4+ cell progression and survival in untreated individuals with HIV-1 infection. AIDS. 2017;31(8):1073-82. doi: 10.1097/QAD.0000000000001437.

6. Touloumi G, Pantazis N, Babiker AG, Walker SA, Katsarou O, Karafoulidou A, et al. Differences in HIV RNA levels before the initiation of antiretroviral therapy among 1864 individuals with known HIV-1 seroconversion dates. AIDS. 2004;18(12):1697-705.

7. Dunn D, Woodburn P, Duong T, Peto J, Phillips A, Gibb D, et al. Current CD4 cell count and the short-term risk of AIDS and death before the availability of effective antiretroviral therapy in HIV-infected children and adults. J Infect Dis. 2008;197(3):398-404. doi: 10.1086/524686.

8. Todd J, Glynn JR, Marston M, Lutalo T, Biraro S, Mwita W, et al. Time from HIV seroconversion to death: a collaborative analysis of eight studies in six low and middle-income countries before highly active antiretroviral therapy. AIDS. 2007;21(Suppl 6):S55-63. doi: 10.1097/01.aids.0000299411.75269.e8.

9. Collaborative Group on AIDS Incubation and HIV Survival. Time from HIV-1 seroconversion to AIDS and death before widespread use of highly-active antiretroviral therapy: a collaborative re-analysis. Lancet. 2000;355(9210):1131-7. doi: 10.1016/S0140-6736(00)02061-4.

10. Pantazis N, Morrison C, Amornkul PN, Lewden C, Salata RA, Minga A, et al. Differences in HIV natural history among African and non-African seroconverters in Europe and seroconverters in Sub-Saharan Africa. PLoS One. 2012;7(3):e32369. doi: 10.1371/journal.pone.0032369.

11. Cori A, Pickles M, van Sighem A, Gras L, Bezemer D, Reiss P, et al. CD4+ cell dynamics in untreated HIV-1 infection: overall rates, and effects of age, viral load, sex and calendar time. AIDS. 2015;29(18):2435-46. doi: 10.1097/qad.0000000000000854. PubMed PMID: 26558543.

12. Johnson LF, May MT, Dorrington RE, Cornell M, Boulle A, Egger M, et al. Estimating the impact of antiretroviral treatment on adult mortality trends in South Africa: A mathematical modelling study. PLoS Med. 2017;14(12):e1002468. doi: 10.1371/journal.pmed.1002468.

13. United Nations Department of Economic and Social Affairs Population Division. World Population Prospects 2019: Volume 1: Comprehensive Tables. 2019.

14. Marston M, Todd J, Glynn JR, Nelson KE, Rangsin R, Lutalo T, et al. Estimating 'net' HIV-related mortality and the importance of background mortality rates. AIDS. 2007;21:S65-S71. doi: 10.1097/01.aids.0000299412.82893.62.

15. Cox DR, Oakes D. Analysis of Survival Data: Chapman and Hall/CRC; 1984.

16. Eaton JW, Brown T, Puckett R, Glaubius R, Mutai KK, Bao L, et al. The Estimation and Projection Package Age-Sex Model and the R-hybrid model: new tools for estimating HIV incidence trends in sub-Saharan Africa. AIDS. 2019;33(Suppl 3):S235-S44. doi: 10.1097/QAD.0000000000002437.

17. Joint United Nations Programme on HIV/AIDS (UNAIDS). Fact sheet - Global AIDS Update 2019. Geneva: UNAIDS, 2019.

18. The IeDEA and COHERE Cohort Collaborations. Global trends in CD4 cell count at the start of antiretroviral therapy: Collaborative study of treatment programs. Clin Infect Dis. 2018;66(6):893-903. doi: 10.1093/cid/cix915.

19. Gelman A, Carlin JB, Stern HS, Dunson DB, Vehtari A, Rubin DB. Bayesian Data Analysis, Third Edition: Chapman and Hall/CRC; 2013.
