# Supplementary material for: Disease progression and mortality with untreated HIV infection: evidence synthesis of HIV seroconverter cohorts, antiretroviral treatment clinical cohorts and population‐based survey data
Source: J Int AIDS Soc. 2021 Sep 21;24(Suppl 5):e25784. doi: 10.1002/jia2.25784 (PMC8454684; doi:10.1002/jia2.25784)
Supplement: Supplementary file 2 [file JIA2-24-e25784-s004.docx]

Appendix S2: Comparison of natural history parameter estimates to previous Spectrum inputs.


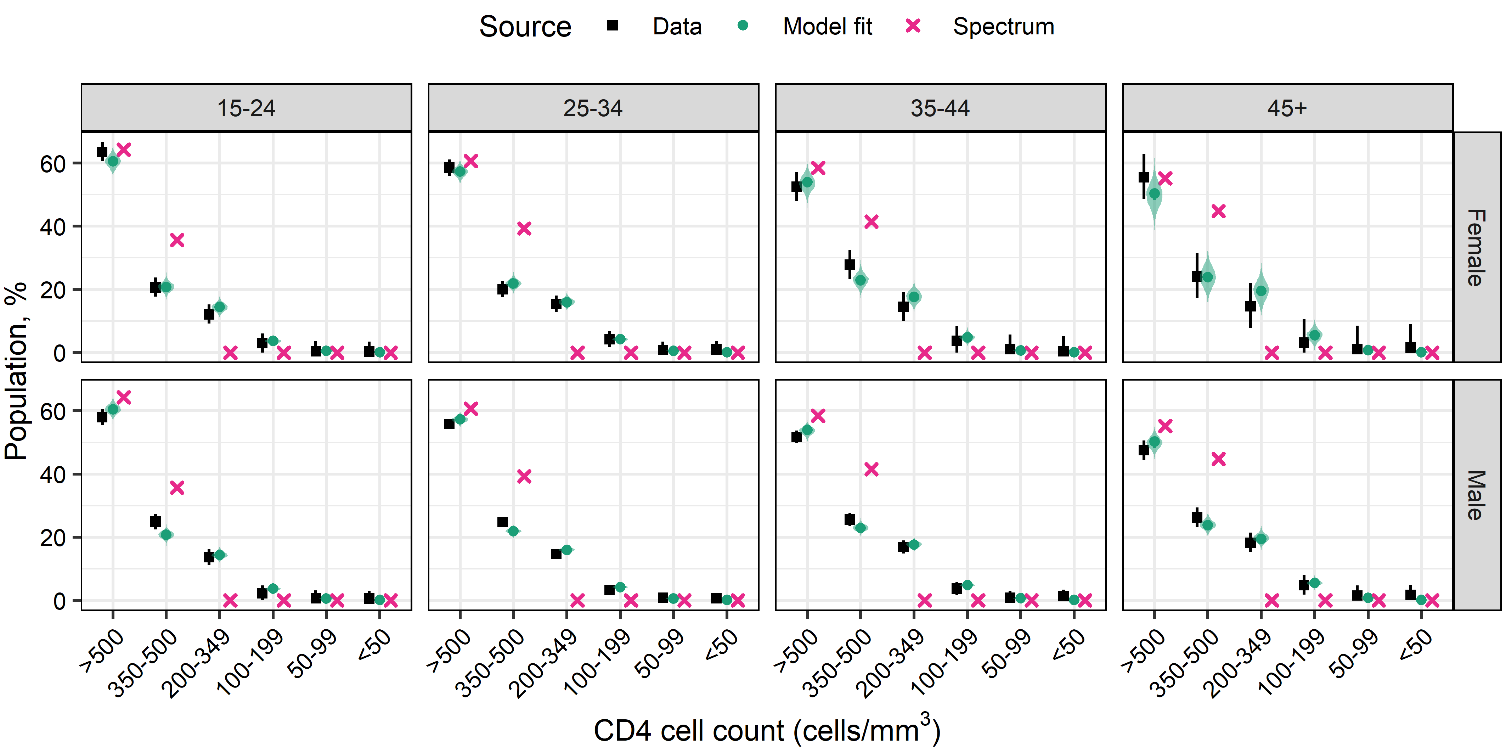


**Figure S1. Initial CD4 cell count distribution.** Black squares with error bars show summary statistics (point estimates and multinomial 95% confidence intervals) calculated from seroconverter counts compiled in Reference [[1](#_ENREF_1)]. Green points, error bars, and shaded regions show posterior mode point estimates, 95% credible intervals, and posterior predictive distributions, respectively. Red x’s show previous Spectrum input assumptions.


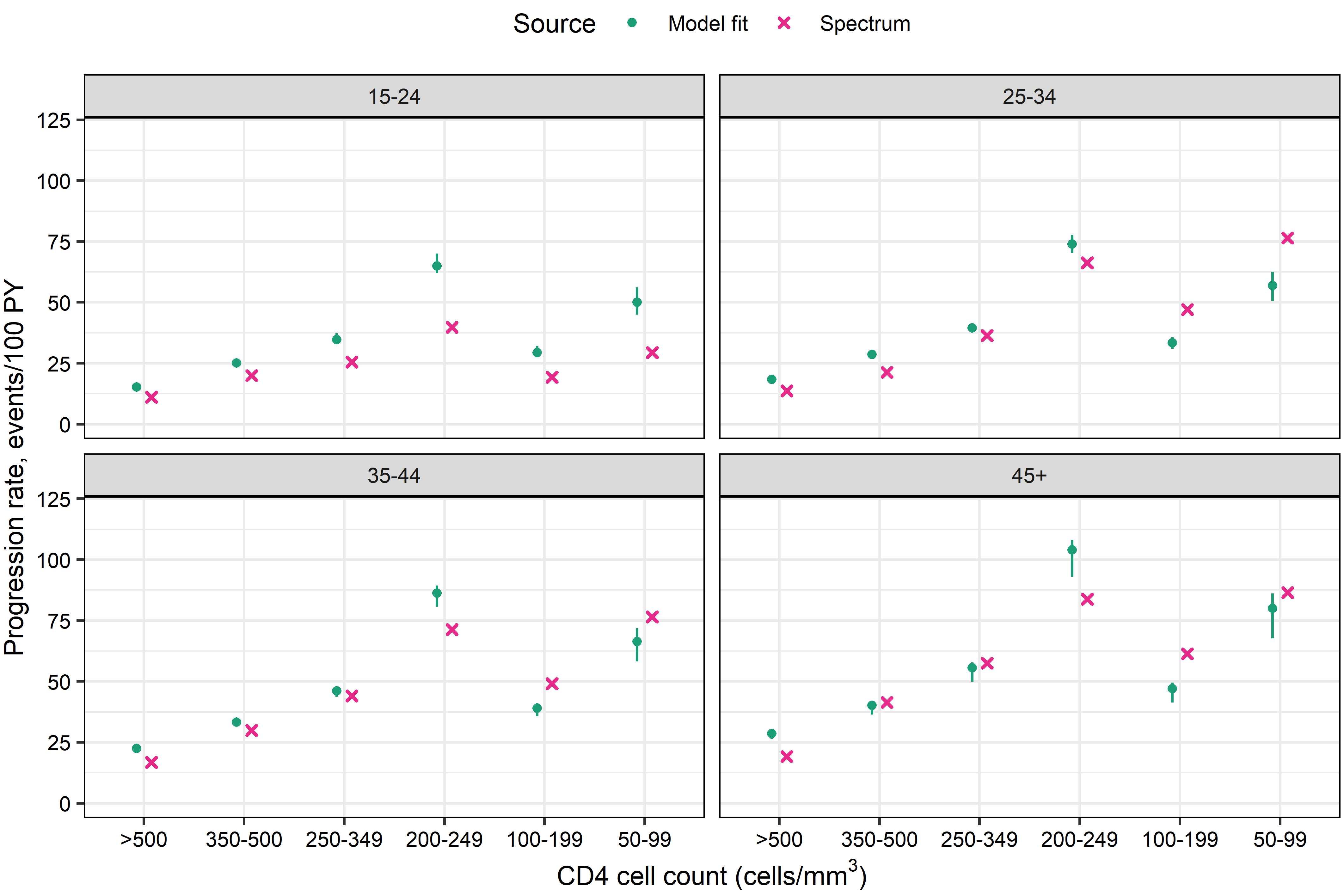


**Figure S2. HIV disease progression rates.** Green points and error bars show posterior mode parameter estimates and 95% credible intervals, respectively. Red x’s show previous Spectrum input assumptions.


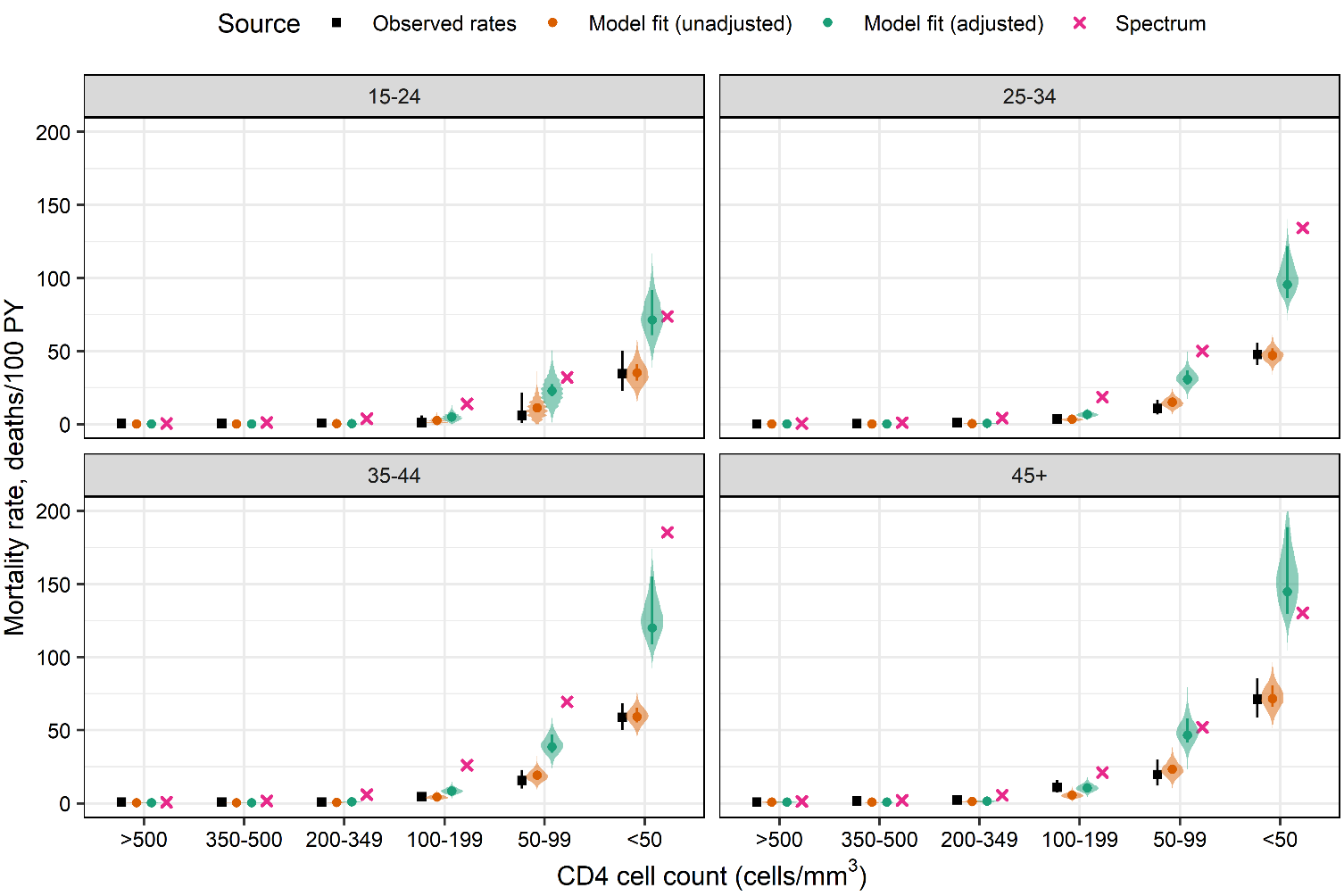


**Figure S3. All-cause mortality rates among people living with HIV.** Black squares show rates reported by Dunn *et al*. [[2](#_ENREF_2)] with Poisson 95% confidence intervals calculated from reported numbers of deaths and person-years of observation. Orange points, error bars, and shaded regions show posterior mode estimates, 95% credible intervals, and posterior predictive distributions, respectively, when the mortality mortality rate ratio is fixed at $\varphi_{4}=1$. Green points, error bars, and shaded regions show posterior mode estimates 95% credible intervals, and posterior predictive distributions using estimated values of $\varphi_{4}$. Red x’s show previous Spectrum input assumptions.


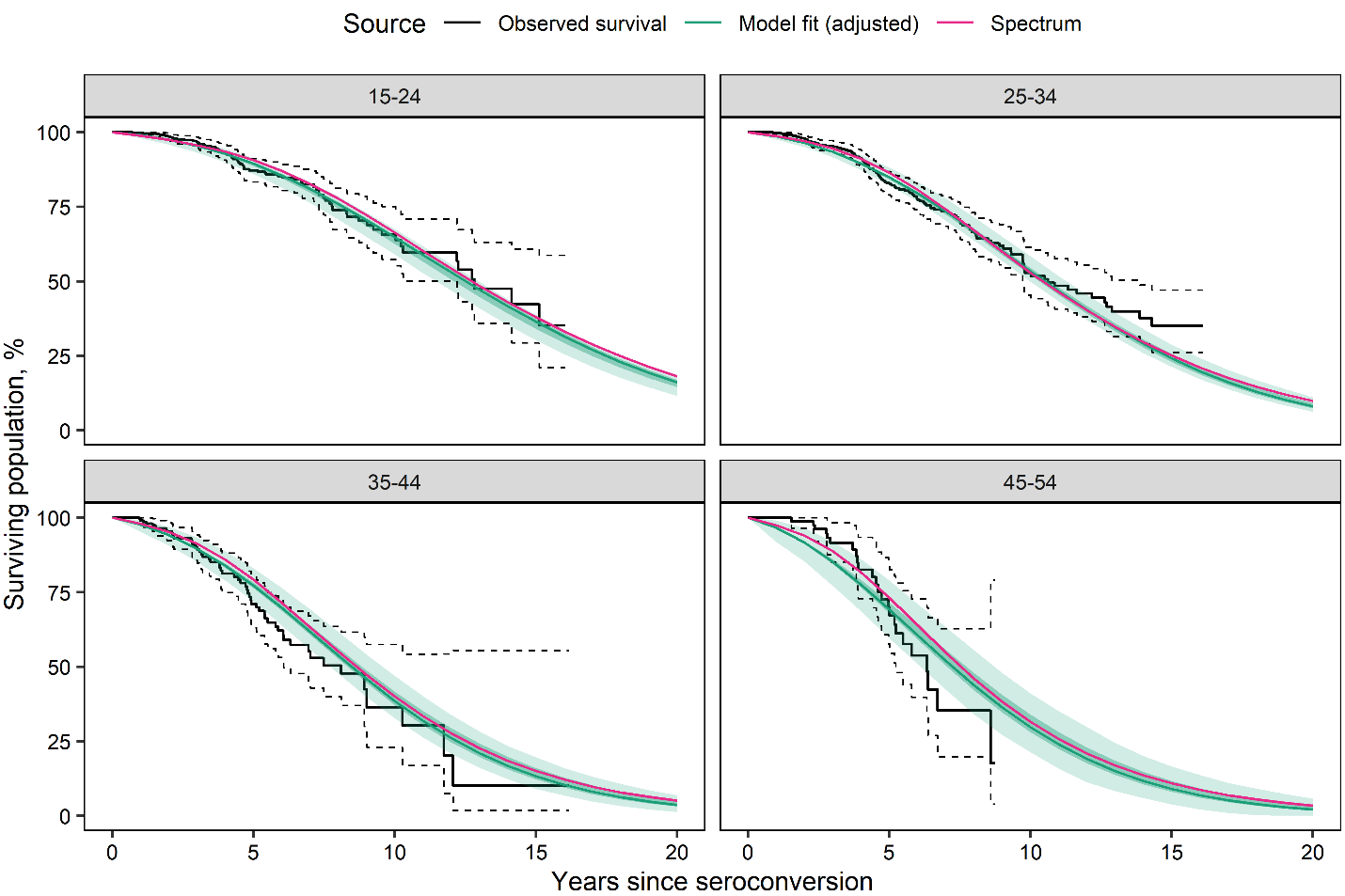


**Figure S4. Survival after seroconversion.** Black curves show Kaplan-Meier survival curves calculated from times from four East African cohorts [[3](#_ENREF_3)] (“observed survival”), with 95% confidence intervals as dashed lines. Solid green curves show posterior mode estimates. Shaded green regions show 95% credible intervals (narrow, darker regions) and 95% posterior predictive intervals (wider, lighter regions). The red curve shows calculated from Spectrum’s previous inputs.

**REFERENCES**

1. Mangal TD, the UNAIDS Working Group on CD4 Progression and Mortality Among Seroconverters, the CASCADE Collaboration in EuroCoord. Joint estimation of CD4+ cell progression and survival in untreated individuals with HIV-1 infection. AIDS. 2017;31(8):1073-82. doi: 10.1097/QAD.0000000000001437.

2. Dunn D, Woodburn P, Duong T, Peto J, Phillips A, Gibb D, et al. Current CD4 cell count and the short-term risk of AIDS and death before the availability of effective antiretroviral therapy in HIV-infected children and adults. J Infect Dis. 2008;197(3):398-404. doi: 10.1086/524686.

3. Todd J, Glynn JR, Marston M, Lutalo T, Biraro S, Mwita W, et al. Time from HIV seroconversion to death: a collaborative analysis of eight studies in six low and middle-income countries before highly active antiretroviral therapy. AIDS. 2007;21(Suppl 6):S55-63. doi: 10.1097/01.aids.0000299411.75269.e8.
