# Supplementary material for: Disease progression and mortality with untreated HIV infection: evidence synthesis of HIV seroconverter cohorts, antiretroviral treatment clinical cohorts and population‐based survey data
Source: J Int AIDS Soc. 2021 Sep 21;24(Suppl 5):e25784. doi: 10.1002/jia2.25784 (PMC8454684; doi:10.1002/jia2.25784)
Supplement: Supplementary file 3 [file JIA2-24-e25784-s001.docx]

## Appendix S3

**CASCADE Collaboration**: Ethics approval has been granted by the following committees: Austrian HIV Cohort Study: Ethik-Kommission der Medizinischen Universität Wien, Medizinische Universität Graz – Ethikkommission, Ethikkommission der Medizinischen Universität Innsbruck, Ethikkommission des Landes Oberösterreich, Ethikkommission für das Bundesland Salzburg; PHAEDRA cohort: St Vincent's Hospital, Human Research Ethics Committee; Southern Alberta Clinic Cohort: Conjoint Health Research Ethics Board of the Faculties of Medicine, Nursing and Kinesiology, University of Calgary; Aquitaine Cohort: Commission Nationale de l'Informatique et des Libertés; French Hospital Database: Commission nationale de l'informatique et des libertés CNIL; French PRIMO Cohort: Comite Consultatif de Protection des Personnes dans la Recherché Biomedicale; SEROCO Cohort: Commission Nationale de l'Informatique et des Libertés (CNIL); German HIV-1 Seroconverter Study: Charité, University Medicine Berlin; AMACS: Bioethics & Deontology Committee of Athens University Medical School and the National Organization of Medicines; Greek Haemophilia Cohort: Bioethics & Deontology Committee of Athens University Medical School and the National Organization of Medicines; ICoNA cohort: San Paolo Hospital Ethic Committee; Italian Seroconversion Study: Comitato etico dell’Istituto Superiore di Sanità; Amsterdam Cohort Studies in Homosexual Men and IDUs: Academic Medical Centre, University of Amsterdam; Oslo and Ulleval Hospital Cohorts: Regional komite for medisinsk forskningsetikk - Øst- Norge (REK 1); Badalona IDU Hospital Cohort: Comité Ético de Investigación Clínica del Hospital Universitari Germans Trias i Pujol; CoRIS-scv: Comité Ético de Investigación Clínica de La Rioja; Madrid Cohort: Ethics Committee of Universidad Miguel Hernandez de Elche; Valencia IDU Cohort: Comité Etico de Investigación Clínica del Hospital Dr. Peset-Valencia; Swiss HIV Cohort Study: Kantonale Ethikkommission, spezialisierte Unterkommission Innere Medizin, Ethikkommission beider Basel, Kantonale Ethikkommission Bern, Comité départemental d'éthique de médecine et médecine communautaire, Commission d'éthique de la recherche clinique, Université de Lausanne, Comitato etico cantonale, Ethikkommission des Kantons St.Gallen; UK Register of HIV Seroconverters: South Birmigham REC; Early Infection Cohorts: Kenya Medical Research Institute, Kenyatta National Hospital, Uganda Virus Research Institute Science and Ethics Committee, Uganda National Council for Science and Technology, Uganda Virus Research Institute Science and Ethics Committee, Uganda National Council for Science and Technology, University of Zambia Research Ethics Committee, Emory IRB, National Ethics Committee of Rwanda, University of Cape Town Research Ethics Committee, University of Kwazulu Natal Nelson R Mandela School of Medicine; Genital Shedding Study Cohort: University Hospitals of Cleveland, IRB for Human Investigation (CWRU), AIDS Research Committee (ARC), STD/AIDS Control Programme, Uganda Ministry of Health, Committee on Human Research (CHR), Office of Research Administration (UCSF), Biomedical Research & Training Institute (BRTI) – Zimbabwe, Institutional Review Office, Fred Hutchinson Cancer Research Center, Medical Research Council of Zimbabwe (MRCZ).
