# Supplementary material for: Disease progression and mortality with untreated HIV infection: evidence synthesis of HIV seroconverter cohorts, antiretroviral treatment clinical cohorts and population‐based survey data
Source: J Int AIDS Soc. 2021 Sep 21;24(Suppl 5):e25784. doi: 10.1002/jia2.25784 (PMC8454684; doi:10.1002/jia2.25784)
Supplement: Supplementary file 4 — Table S1. Model sensitivity to sex differences in progression and mortality. [file JIA2-24-e25784-s003.docx]

**Table S1. Model sensitivity to sex differences in progression and mortality.**

|  | **Model without sex differences** | **Model with sex differences** |
| --- | --- | --- |
| Parameters, # | 11 | 12 |
| Log-Likelihood of posterior mode | -12,175.5 | -12,175.1 |
| Deviance Information Criterion [[1](#_ENREF_1)] | 24,367.3 | 24,363.7 |
| **Initial CD4 cell count** |  |  |
| Shape parameter ($\psi_{1}$) | 1.92 (1.86-1.99) | 1.93 (1.86-1.98) |
| Age 15-24 median, CD4 cells/mm^3^ ($\psi_{2}$) | 579 (570-589) | 578 (571-588) |
| Age effect ($\psi_{3}$) | 0.044 (0.036-0.055) | 0.042 (0.037-0.055) |
| **CD4 cell count trend** |  |  |
| Shape parameter ($\theta_{1}$) | 0.31 (0.22-0.43) | 0.35 (0.24-0.42) |
| Age 15-24 CD4 cell depletion time, years ($\theta_{2}$) | 23.2 (21.5-24.4) | 23.0 (21.3-23.8) |
| Age effect ($\theta_{3}$) | 0.133 (0.104-0.142) | 0.125 (0.105-0.135) |
| **HIV-related mortality** |  |  |
| Shape parameter ($\varphi_{1}$) | 0.977 (0.975-0.979) | 0.977 (0.976-0.979) |
| Age 15-24 mortality rate at 0 CD4 cells/mm^3^, person-year^-1^ ($\varphi_{2}$) | 0.59 (0.51-0.69) | 0.55 (0.51-0.66) |
| Age effect ($\varphi_{3}$) | 0.34 (0.23-0.50) | 0.40 (0.25-0.46) |
| Mortality rate ratio ($\varphi_{4}$) | 2.03 (1.83-2.60) | 2.18 (1.84-2.40) |
| **Rate ratio of progression and mortality in females relative to males**^†^ | not included | 0.99 (0.94-1.02) |
| **ART allocation weight** ($\omega$) | 0.21 (0.16-0.25) | 0.20 (0.18-0.25) |

Posterior mode parameter point estimates (95% credible intervals) are shown.

^†^ We applied the same sex ratio to disease progression and mortality rates.

**REFERENCES**

1. Gelman A, Carlin JB, Stern HS, Dunson DB, Vehtari A, Rubin DB. Bayesian Data Analysis, Third Edition: Chapman and Hall/CRC; 2013.
